# Signaling Pathway Analysis and Downstream Genes Associated with Disease Resistance Mediated by *GmSRC7*

**DOI:** 10.3390/plants15020318

**Published:** 2026-01-21

**Authors:** Aoga Li, Chongyang Yao, Ting Yan, Xiaomin Hao, Dongying Geng, Qi Zhang, Hui Li, Wenquan Bao, Yue Bai

**Affiliations:** 1 School of Forestry, Inner Mongolia Agricultural University, Hohhot 010018, China; gengdongying@emails.imau.edu.cn (D.G.); 15931898338@163.com (Q.Z.); huil1984@imau.edu.cn (H.L.); bwq@imau.edu.cn (W.B.); 2 School of Life Sciences, Inner Mongolia University, Hohhot 010070, China; yaochongyang2025@163.com (C.Y.); hxmin0825@imu.edu.cn (X.H.); 3 Institute of Genetics and Breeding, Inner Mongolia Academy of Forestry, Hohhot 010010, China; nmgyanting@163.com

**Keywords:** soybean mosaic virus, tobacco mosaic virus, *R* gene, downstream signaling pathway, salicylic acid, *GmSRC7*, immune pathway

## Abstract

*GmSRC7* is a broad-spectrum antiviral *R* gene from soybean, but its downstream and functionally related genes remain unclear. Virus-induced gene silencing (VIGS) assays in *Nicotiana benthamiana* (*Nb*) showed that suppression of several gene families—WRKY transcription factors, chaperones, ethylene pathway components, MAPK cascade elements, salicylic acid (SA) signaling genes, calcium-dependent protein kinases, nuclear migration proteins, RNA replication-related genes, and immune regulators—consistently weakened *GmSRC7*-mediated resistance to Soybean Mosaic Virus (SMV) and Tobacco Mosaic Virus (TMV). Targeted silencing of four regulatory genes—*NbEDS1*, *NbARF1*, *NbSGT1*, and *NbCOI1*—markedly enhanced *GmSRC7*-mediated resistance to SMV and TMV in our experiments. Silencing the serine/threonine kinase gene *NbPBS1* increased *GmSRC7*-conferred resistance to SMV but did not significantly alter its resistance to TMV. Transient expression assays showed that *NbARF1*, *NbSGT1*, and *NbCOI1* antagonize *GmSRC7*-mediated defense against SMV and TMV, whereas *NbPBS1* specifically suppresses anti-SMV activity without affecting TMV resistance. Transient overexpression of SA-degrading enzymes (*AtS3H*, *AtS5H*, and *NahG*) significantly reduced *GmSRC7*-conferred resistance to SMV, indicating that SA is essential for this R protein-mediated defense. Genes were also grouped by immune pathways and function: co-expression of chaperone family genes inhibited *GmSRC7* activity against SMV and TMV, while co-expression of WRKY family genes enhanced anti-SMV activity of *GmSRC7*. Finally, transient silencing of soybean genes *GmEDS1*, *GmSGT1-1*, *GmSGT1-2*, *GmJAR1*, and *GmSGS3* compromised *GmSRC7*-mediated resistance to SMV.

## 1. Introduction

Soybean (*Glycine max*) is an annual herbaceous legume. It is vulnerable to multiple viral infections, among which SMV is the most widespread and damaging to seed quality and yield [1]. SMV, a member of the Potyviridae family, spreads mainly via infected seed and by soybean aphids. Infection produces characteristic symptoms, such as leaf necrosis and mosaic patterns, and affects both wild and cultivated soybean varieties [2,3,4]. Owing to the diversity of soybean cultivars, SMV has evolved many distinct serotypes. In the United States, SMV strains are grouped into seven serotypes (G1–G7) based on virulence against soybean lines, while in China they are classified into 22 serotypes (SC1–SC22) according to pathogenicity [5,6,7].

Plants possess a two-layered immune system. The first layer, known as pattern-triggered immunity (PTI), employs surface receptors to identify common pathogen molecules. In response, pathogens release effector proteins to suppress PTI. However, plants have developed a second defense layer: resistance (R) proteins that detect these effectors and initiate a more robust defense called effector-triggered immunity (ETI) [8,9,10]. Research suggests that soybean resistance to SMV is governed by complex gene families, with multiple independent resistance loci identified for various SMV strains, most of which encode the R protein [11]. Thus, the *R* gene-mediated disease resistance pathway in soybean against SMV is particularly vital. Typically, R proteins are intracellular multi-domain receptors characterized by nucleotide-binding sites and leucine-rich repeat (NLR) motifs [12]. These proteins feature an N-terminal coiled-coil (CC) or Toll/IL-1 receptor (TIR) domain, a central nucleotide-binding (NB) region, and a C-terminal leucine-rich repeat (LRR) sequence [13]. NLR proteins can be categorized as CC–NLR (CNL) or TIR–NLR (TNL) based on their N-terminal structures.

Numerous downstream genes in *R*-gene-mediated signaling pathways have been identified. For example, the *RAR1* (Required for Mla 12 Resistance) gene, initially discovered in barley, is crucial for *MLA6* and *MLA12*-mediated resistance to *powdery mildew* [14]. Research has shown that the RAR1–SGT1 complex is vital for RPS5-mediated disease resistance [15]. Additionally, the *NDR1* gene, cloned from *Arabidopsis thaliana*, plays a role in *R*-gene-mediated defense against bacteria and fungi [16]. In 2003, Takahashi A. et al. identified Heat Shock Protein 90 (HSP90) as another RAR1-interacting protein through yeast two-hybrid experiments, demonstrating its necessity for RPS2-mediated resistance to *Pseudomonas syringae* [17]. Furthermore, *EDS1*, identified in 1996, is linked with the *RPP* family resistance genes in *A*. *thaliana*. Studies later revealed that *EDS1* and *NDR1* are involved in resistance mediated by *RPS2*, *RPM1*, and *RPS5*. *EDS1*, possessing lipid hydrolytic activity, functions upstream of the SA-mediated *PR1* gene [18,19,20] and is essential for TMV resistance mediated by the *N* gene [21]. Recent findings indicate that EDS1 operates upstream of NRG1 in TIR–NLR-mediated defense responses [22]. The EDS1–SAG101–NRG1 regulatory network is well-documented in TNL-mediated disease defense responses [23,24]. PAD4 (Phytoalexin-deficient 4), closely related to EDS1, functions upstream in the SA signaling pathway [25]. The roles of GmEDS1 and GmPAD4 in soybean SMV immunity were first reported in 2012 [26]. EDR1 (Enhanced Disease Resistance 1), a MAPKK kinase, regulates immune responses mediated by mitogen-activated protein (MAP) kinase, negatively affecting the MKK4/MKK5-MPK3/MPK6 signaling pathway in *A*. *thaliana* [27,28,29]. Silencing the MAPK signaling pathway genes *GmMAPK4* and *GmMAPK6* in soybean enhances host resistance to SMV [30]. *JAR1* (Jasmonic Responsive 1), discovered in 1998, influences bacterial resistance in *A*. *thaliana* [31]. The WRKY30 transcription factor enhances Cucumber Mosaic Virus (CMV) resistance in plants [32].

Molecular biology techniques have identified numerous soybean host genes linked to SMV resistance [33]. GmAKT2 is involved in regulating K^+^ transport in the phloem, enhancing the redistribution of K^+^ in soybean plants, and increasing its concentration in young leaves [34]. GmPP2C acts as a key regulatory factor in *Rsv3*-mediated resistance responses [35]. In model plants like *Arabidopsis* and *N*. *benthamiana*, MAPK is crucial for disease resistance. GmMPK4s negatively regulate SA accumulation and defense responses but positively influence plant growth and development [36,37]. Silencing *GmHSP40.1* raises the likelihood of SMV infection [38]. Soybean GmCNX1 shares high amino acid sequence similarity with CNX1 proteins in *Medicago*, *Cucumis*, and *Arabidopsis*, and its overexpression enhances resistance to various SMV strains [39]. In *Rsv1* genotype soybeans, SMV-G7 strain infection significantly upregulates *eIF5A* (Eukaryotic translation initiation factor 5A) expression. Plants with silenced *GmeIF5A* show significantly reduced post-infection necrosis symptoms and LSHR (Lethal systemic hypersensitive response) [40]. eEF1A (Eukaryotic elongation factor 1A), a known host factor in viral pathogenesis, interacts with SMV, and silencing *GmeEF1A* inhibits SMV accumulation [41]. The expression level of *miR168* significantly increases in soybeans with the *Rsv1* resistance gene when infected by the SMV-G7 strain. Overexpressing the RNAi pathway component *SGS3* inhibits *miR168* and partially restores the AGO1 protein, reducing SMV infection symptoms [42]. Additionally, *GmEDR*1, *GmEDS1*, *GmJAR1*, *GmHSP90*, and *GmPAD4* are essential components in *Rsv1*-mediated resistance [43].

*GmSRC7* provides resistance not only to SMV but also to TMV in *N*. *benthamiana*. TMV, a model single-stranded RNA virus, specifically infects plants like *tobacco*, *tomato*, and other *solanaceous* species [44,45]. Numerous genes involved in TMV resistance have been identified in *tobacco*. Key components of the *N*-gene-mediated resistance to TMV include *NbRAR1*, *NbSGT1*, and *NbSCF* [46,47,48,49]. Silencing genes such as *NbSKP1*, *NbMEK2*, *NbWRKY1*, *NbWRKY2*, *NbWRKY3*, *NbCOI1* (Coronatine Insensitive 1), *NbAOX1* (Alternative Oxidase 1), and *ARF1* (ADP-ribosylation Factor 1) disrupt this resistance [50,51,52,53,54,55,56,57,58,59]. Silencing *NbWIPK* or *NbSIPK* slightly reduces viral replication, increases SA levels, and decreases jasmonic acid (JA) concentrations [60]. Research indicates that suppressing *NbCTR1* expression accelerates the HR mediated by the *N*-gene against TMV [61]. In *tomatoes*, recent studies show that EREBP proteins directly interact with Pto kinase, enhancing *R*-gene-mediated resistance to *Pseudomonas syringae* [62,63]. NbRdRP1m, an RNA-dependent RNA polymerase (RdRP), is activated by SA or induced by TMV, aiding in resistance to TMV infection [64]. *NbNUDC* encodes a nuclear migration protein that relies on regulatory components like microtubules, kinesin motor complexes, LIS1, and NUDC for movement [65]. *TpoxC1* (*Tobacco* Peroxidase Gene 1) encodes a peroxidase kinase and positively regulates the *tobacco* *N*-gene-mediated self-defense system [66]. *PBS1* (AvrPphB Susceptible Protein 1) encodes a highly conserved serine/threonine protein kinase in flowering plants. In *Arabidopsis thaliana*, PBS1 is cleaved by the AvrPphB (Pseudomonas phaseolicola B) protease, which activates *RPS5* and triggers HR [67,68].

Salicylic acid (SA) is a crucial plant hormone that transmits defense signals both locally and systemically. In plants, NPR1 serves as a receptor for SA, positively regulating the SA signaling pathway, while *NPR3* and *NPR*4 function as antagonists [69]. SA is widely recognized as a signaling molecule in plant–pathogen interactions [70]. Research indicates that 90% of SA is synthesized via the isochorismate synthase (ICS) pathway, with only 10% produced through the phenylalanine ammonia-lyase (PAL) pathway [71]. In this process, chorismate is converted by ICS proteins into isochorismate, which is then transported to the cytoplasm by the EDS5 (Enhanced Disease Susceptibility 5) protein and catalyzed by PBS3 (AvrPhB Susceptible 3) to bind with L-glutamate, forming isochorismoyl-glutamate A [72]. *Arabidopsis* mutants *pbs3* and *eps1* exhibit reduced SA accumulation and increased susceptibility to bacterial pathogens, highlighting that *AtPBS3* and *AtEPS1* are genes involved in SA metabolic pathways [73].

In our preliminary work, we identified a soybean virus-resistance gene, *GmSRC7* (SMV Resistance Cluster), that is homologous to the *tobacco* *N* gene and confers broad-spectrum resistance. The functional roles of its associated proteins and downstream signaling pathways remain unclear. This study aimed to determine which known R-protein signaling pathway members mediate *GmSRC7* function.

## 2. Results

### 2.1. Screening of Functionally Related Genes of GmSRC7 in Soybeans

Several host soybean genes implicated in resistance to SMV have been reported previously; these genes were identified in earlier studies [33] and include *GmAKT2*, *GmPP2C*, *GmMPK6*, *GmMPK4*, *GmHSP40.1*, *GmHSP90*, *GmCNX1*, *GmeIF5A*, *GmeEF1*, *GmAGO1*, *GmSGS3*, *GmRAR1*, *GmSGT1-1*, *GmSGT1-2*, *GmEDS1*, *GmEDR1*, *GmJAR1*, *GmPAD4*, *GmWRKY6*, and *GmWRKY30* (Supplementary Table S1). Because these twenty genes play central roles in soybean resistance to SMV, we examined their involvement in the *GmSRC7*-mediated anti-SMV immune response.

**Figure 1:**
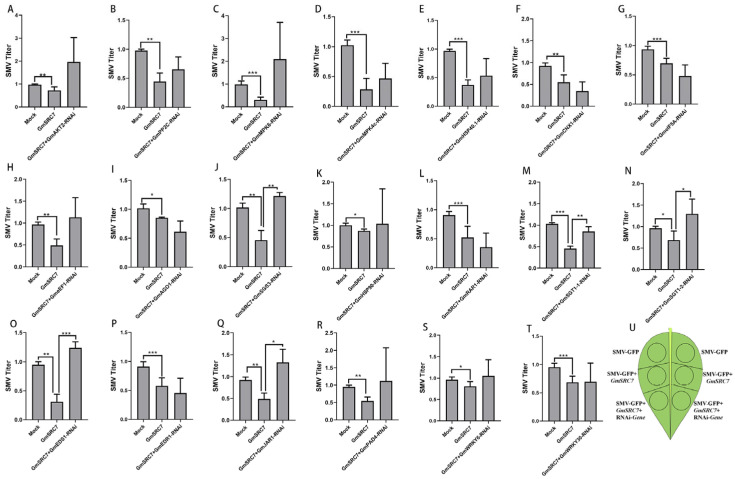
Detection of SMV titer after transient overexpression of *GmSRC7* and transient silencing of candidate genes in soybean leaves using leaf injection. Panels show silencing of (**A**) *GmAKT2*, (**B**) *GmPP2C*, (**C**) *GmMPK6*, (**D**) *GmMPK4*, (**E**) *GmHSP40.1*, (**F**) *GmHSP90*, (**G**) *GmCNX1*, (**H**) *GmeIF5A*, (**I**) *GmeEF1*, (**J**) *GmAGO1*, (**K**) *GmSGS3*, (**L**) *GmRAR1*, (**M**) *GmSGT1-1*, (**N**) *GmSGT1-2*, (**O**) *GmEDS1*, (**P**) *GmEDR1*, (**Q**) *GmJAR1*, (**R**) *GmPAD4*, (**S**) *GmWRKY6*, and (**T**) *GmWRKY30*. (**U**) Schematic of the co-expression design: each leaf was injected at six sites within a defined circle. SMV-GFP+*GmSRC7* indicates co-expression of SMV and *GmSRC7*, and SMV+*GmSRC7*+RNAi denotes co-expression of SMV-GFP and *GmSRC7* together with the RNAi construct targeting the indicated gene. The SMV inoculum concentration was identical for all injection sites. The *t*-test was performed between samples in different treatment groups. * *p* < 0.05; ** *p* < 0.01, *** *p* < 0.001.

To identify genes functionally related to *GmSRC7* and its downstream signaling components, candidate genes were cloned into silencing vectors and transiently silenced in soybean leaves using an *Agrobacterium*-mediated overexpression/silencing system. SMV titers were measured during SMV-GFP expression, *GmSRC7*+SMV-GFP co-expression, and *GmSRC7*+SMV-GFP+RNAi-gene co-expression (Figure 1U). (SMV-GFP is a 35S-driven infectious clone carrying the SMV genome and a GFP tag, and co-expression refers to simultaneous infiltration of *Agrobacterium* cultures harboring the respective vectors into soybean leaves.) By comparing SMV titers at the *Agrobacterium* infiltration sites, we identified genes functionally associated with *GmSRC7*.

SMV successfully infected soybean leaves, and silencing produced the expected downregulation of the assayed genes (Supplementary Figure S1). In soybean, *GmSRC7* confers resistance to SMV. Overexpression of *GmSRC7* suppressed SMV accumulation, whereas silencing *GmSGT1-1*, *GmSGT1-2*, *GmEDS1*, *GmJAR1*, or *GmSGS3* markedly attenuated *GmSRC7*-mediated resistance, indicating that these genes act downstream of or are functionally linked to *GmSRC7* (Figure 1).

### 2.2. SA Is a Key Hormone That Mediates Resistance

Transcriptome analysis revealed marked changes in SA-related gene expression during SMV infection (Supplementary Figure S2). Affected genes include SA-biosynthesis pathway genes *GmPBS1.1*, *GmPBS1.2*, *GmPBS1.3*, *GmPAL1.1*, *GmPAL1.2*, *GmPAL1.3*, and *GmICS1*; the SA-responsive gene *GmPR1*; and the SA receptor genes *GmNPR1.2* and *GmNPR1.1*. These results suggest that SA is a key hormone in soybean resistance to SMV.

**Figure 2:**
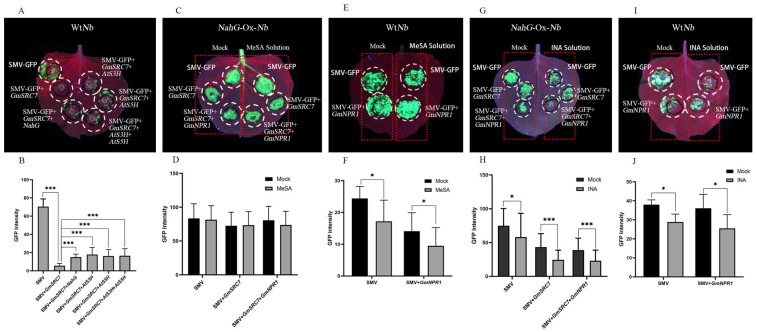
Virus accumulation in infected regions of Wt*Nb* and *NahG*-Ox-*Nb* leaves at day 7. (**A**,**B**) Phenotype and fluorescence intensity of Wt*Nb* leaves co-expressing the constructs. (**C**,**D**) Phenotype and fluorescence intensity of *NahG*-Ox-*Nb* leaves co-expressing the constructs with MeSA treatment. (**E**,**F**) Phenotype and fluorescence intensity of Wt*Nb* leaves co-expressing the constructs with MeSA treatment. (**G**,**H**) Phenotype and fluorescence intensity of *NahG*-Ox-*Nb* leaves co-expressing the constructs with INA treatment. (**I**,**J**) Phenotype and fluorescence intensity of Wt*Nb* leaves co-expressing the constructs with INA treatment. Mock denotes a solvent-free control. The left red box in a single leaf represents the treatment without reagents, while the right red box represents the treatment with reagents. The *t*-test was performed between samples in different treatment groups. * *p* < 0.05; *** *p* < 0.001.

We hypothesized that transient overexpression of *AtS3H*, *AtS5H*, and *NahG* (Supplementary Table S2) in wild-type *Nicotiana benthamiana* (Wt*Nb*) leaves would lower leaf SA levels and thereby alter *GmSRC7*-mediated resistance to SMV. To test this, we transiently overexpressed *NahG*, *AtS3H*, and *AtS5H* in *N*. *benthamiana* leaves and then co-expressed each with *GmSRC7* to measure SMV accumulation. *AtS3H* and *AtS5H* acted synergistically (Figure 2A,B). Overexpression of these genes diminished *GmSRC7*-conferred resistance to SMV, likely because of SA degradation.

To test whether SA contributes to SMV resistance, we assessed the effect of methyl salicylate (MeSA) on *GmSRC7*-mediated resistance in Wt*Nb* and in *NahG*-Ox-*Nb* plants that stably overexpress the *NahG* gene. SMV accumulation was measured during SMV-GFP expression and during *GmSRC7*+SMV-GFP co-expression. The experimental group received MeSA solvent treatment, while the control group comprised SMV-GFP expression and *GmSRC7*+SMV-GFP co-expression without MeSA solvent (Figure 2C,E). The results show that MeSA enhanced resistance in Wt*Nb* plants but not in *NahG*-Ox-*Nb* plants, likely because stable overexpression of *NahG* caused more extensive SA degradation than exogenous application (Figure 2C–F).

GmNPR1 is an SA receptor in soybean that regulates SA-mediated disease resistance and immunity. To test whether its overexpression amplifies the SA response, we expressed *GmNPR1* in *Nb* leaves and applied exogenous MeSA. SMV accumulation was measured during SMV-GFP+*GmNPR1* expression and during SMV-GFP+*GmSRC7*+*GmNPR1* co-expression. The experimental group received MeSA solvent treatment, while the control group comprised SMV-GFP+*GmNPR1* co-expression and SMV-GFP+*GmSRC7*+*GmNPR1* co-expression without MeSA solvent (Figure 2C,E). The results showed that neither exogenous MeSA application nor transient overexpression of *GmNPR1* restored *GmSRC7*-mediated resistance to SMV in the *NahG*-Ox-*Nb* plant (Figure 2C–F). In contrast, in Wt*Nb* plants, exogenous MeSA markedly reduced SMV accumulation, and *GmNPR1* overexpression further enhanced MeSA-induced resistance. Together, these findings indicate that exogenous MeSA induces resistance to SMV and that *GmNPR1* overexpression augments that resistance.

INA (2,6-dichloroisonicotinic), an SA analog, is commonly used to study how exogenous treatments affect plant disease resistance and secondary metabolism. We therefore tested whether exogenous INA application could restore *GmSRC7*-mediated resistance to SMV in *NahG*-Ox-*Nb* plants and whether it could confer SMV resistance in Wt*Nb*. SMV accumulation was measured during SMV-GFP expression, during SMV-GFP+*GmSRC7* co-expression, during SMV-GFP+*GmNPR1* co-expression, and during SMV-GFP+*GmSRC7*+*GmNPR1* co-expression. The experimental group received MeSA solvent treatment, while the control group comprised the same four expression conditions without MeSA solvent (Figure 2G, I).

INA treatment markedly reduced SMV accumulation in Wt*Nb* and *NahG*-Ox-*Nb* plants, and overexpression of *GmNPR1* further enhanced this resistance (Figure 2G–J). In *NahG*-Ox-*Nb* plants, MeSA treatment failed to fully restore *GmSRC7*-mediated resistance to SMV, likely because constitutive *NahG* expression degrades SA more extensively than exogenous application (Figure 2C,D). Overexpression of *GmNPR1* modestly increased *GmSRC7*-mediated resistance to SMV, consistent with *GmSRC7* acting through the SA pathway.

### 2.3. Validation of Downstream and Functionally Related Genes of GmSRC7 in N. benthamiana

Next, we screened downstream and functionally related genes of *GmSRC7* in *N*. *benthamiana*. We identified 36 disease-related genes that may carry out diverse roles in plant growth and development (Supplementary Table S3). For the experiments, we used *GmSRC7*-Ox-*Nb* and included Wt*Nb* as the control. Ten days after silencing 36 candidate genes in *GmSRC7*-Ox-*Nb*, plant phenotypes were recorded. Three to four young upper leaves were then chosen for transient overexpression assays with SMV and TMV. (TMV-GFP is a 35S-driven infectious clone carrying the TMV genome and a GFP tag, and co-expression refers to simultaneous infiltration of *Agrobacterium* cultures harboring the respective vectors into *Nb* leaves.)

**Figure 3:**
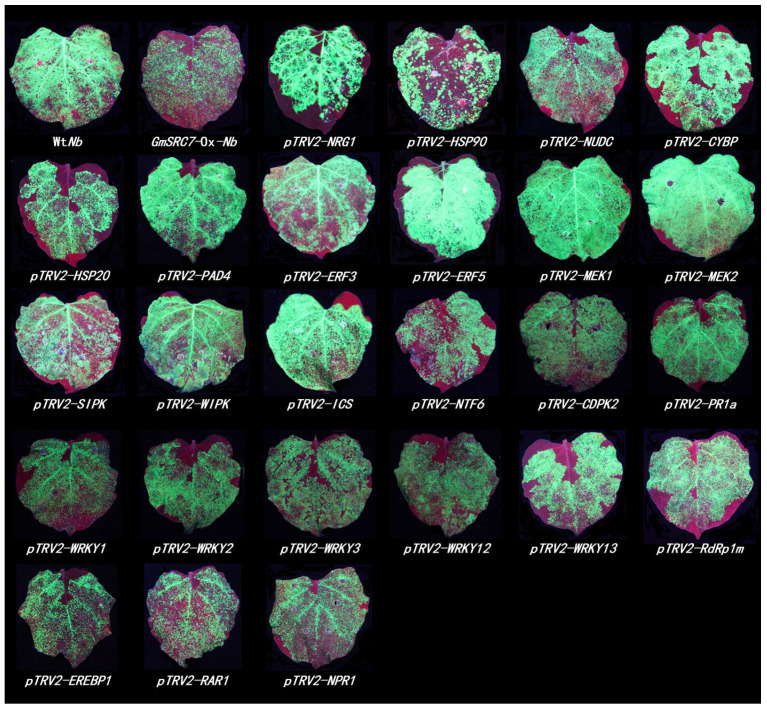
The phenotype image of SMV-GFP infection in *GmSRC7*-Ox-*Nb* plants following VIGS is presented. Wt*Nb* served as the positive control, while *GmSRC7*-Ox-*Nb*, representing the overexpressed *GmSRC7* transgenic *Nicotiana benthamiana*, functioned as the negative control. The experimental group employed the *GmSRC7*-Ox-*Nb* plant. Virus accumulation was detected under UV irradiation 5 days post-SMV-GFP infection, following a 10-day period of target gene silencing via VIGS.

**Figure 4:**
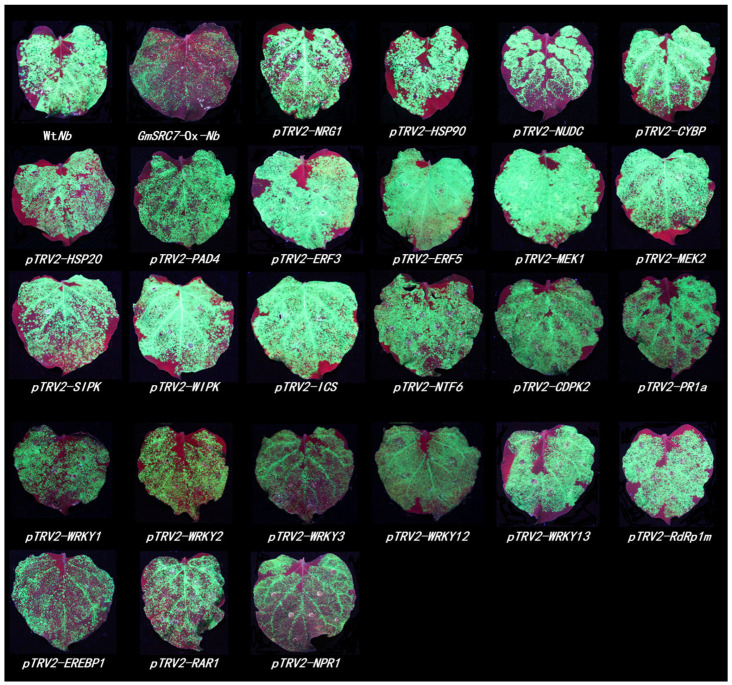
The phenotype image of TMV-GFP infection in *GmSRC7*-Ox-*Nb* plants following VIGS is presented. Wt*Nb* served as the positive control, while *GmSRC7*-Ox-*Nb*, representing the overexpressed *GmSRC7* transgenic *Nicotiana benthamiana*, functioned as the negative control. The experimental group employed the *GmSRC7*-Ox-*Nb* plant. Virus accumulation was detected under UV irradiation 5 days post-TMV-GFP infection, following a 10-day period of target gene silencing via VIGS.

Five days after infection, viral accumulation was visualized under UV light. Using the VIGS system to suppress 25 genes—*NRG1*, *HSP90*, *NUDC*, *CYBP*, *HSP20*, *PAD4*, *ERF3*, *ERF5*, *MEK1*, *MEK2*, *SIPK*, *WIPK*, *ICS*, *NTF6*, *CDPK2*, *PR1a*, *WRKY1*, *WRKY2*, *WRKY3*, *WRKY12*, *WRKY13*, *RdRp1m*, *EREBP1*, *RAR1*, and *NPR1*—resulted in a substantial increase in green fluorescence intensity relative to the empty vector pTRV2. These results indicate that silencing these 25 genes compromises *GmSRC7*-mediated resistance to SMV and TMV (Figure 3 and Figure 4). Silencing six genes—*AOX1*, *TpoxC1*, *RDR6*, *MYB1*, *NPK1*, and *CTR1*—produced no significant change in fluorescence intensity relative to the empty vector pTRV2, indicating no substantial effect on *GmSRC7*-mediated activity against SMV and TMV (Supplementary Figure S3). Notably, interference with five genes—*EDS1*, *COI1*, *PBS1*, *SGT1*, and *ARF1*—increased *GmSRC7* resistance to both viruses, which suggests antagonistic interactions with antiviral activity of *GmSRC7* (Supplementary Figure S4). To assess interference efficiency, we quantified transcript levels by qRT-PCR and found that expression of 10 genes, including *ARF1*, *COI1*, *PBS1*, *ICS*, *MEK1*, *AOX1*, *ERF5*, *TpoxC1*, and *RDR6*, was successfully suppressed (Supplementary Figure S5).

**Figure 5:**
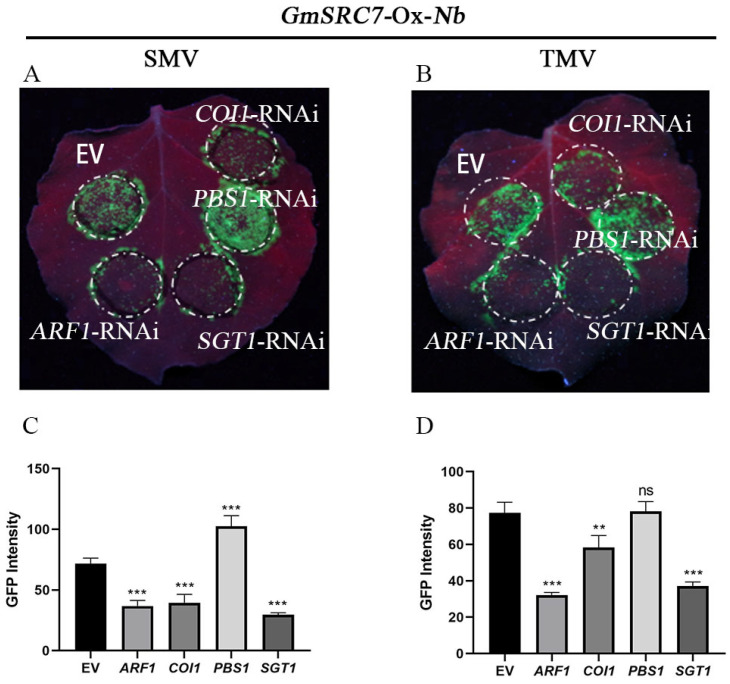
Phenotype and fluorescence intensity of genes that suppressed *GmSRC7*-mediated resistance in the pCB2004B local transient overexpression system. (**A**) Five days after mixed infection with SMV and the recombinant vector containing the target gene, viral accumulation was visualized under UV illumination. (**B**) Five days after mixed infection with TMV and the recombinant vector containing the target gene, viral spread was observed under UV light. (**C**) Fluorescence intensity was quantified five days after mixed infection with SMV and the pCB2004B recombinant vector. (**D**) Fluorescence intensity was quantified five days after mixed infection with TMV and the pCB2004B recombinant vector. EV served as the baseline control. The *t*-test was performed between samples in different treatment groups. ** *p* < 0.01, *** *p* < 0.001. ns, non-significant.

We next examined the roles of *ARF1*, *COI1*, *SGT1*, and *PBS1*. Suppressing *ARF1*, *COI1*, or *SGT1* markedly reduced viral load, indicating that antiviral activity of *GmSRC7* against SMV and TMV was enhanced (Figure 5). By contrast, *PBS1* suppression impaired *GmSRC7*-mediated resistance to SMV but did not significantly affect its TMV resistance. Successful suppression of *ARF1*, *COI1*, *SGT1*, and *PBS1* was confirmed (Supplementary Figure S6).

**Figure 6:**
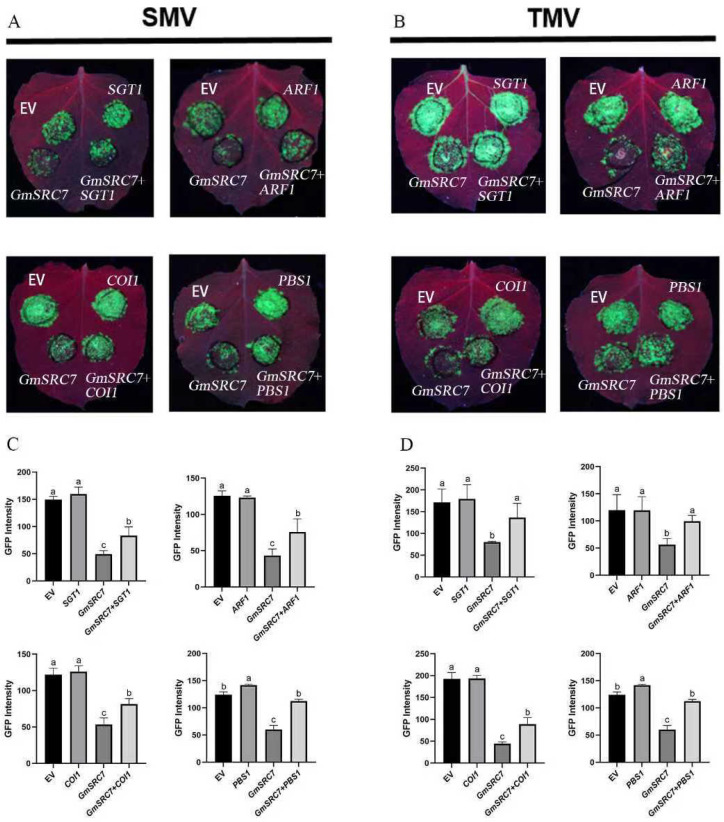
Phenotype and fluorescence quantification of genes that suppress *GmSRC7*-mediated resistance in an overexpression validation system. (**A**) Phenotypes following 5 days of co-expression with SMV. (**B**) Phenotypes following 5 days of co-expression with TMV; includes fluorescence quantification from the transient overexpression assay. (**C**) Fluorescence measured under UV illumination after 5 days of transient co-expression with SMV and overexpression of *ARF1*, *COI1*, *PBS1*, and *SGT1*. (**D**) Fluorescence measured under UV illumination after 5 days of transient co-expression with TMV and overexpression of *ARF1*, *COI1*, *PBS1*, and *SGT1*. The *t*-test was performed between samples in different treatment groups. The same letter indicates no significant difference between groups, while different letters indicate significant differences.

In the VIGS and pCB2004B-mediated local transient interference systems, inhibition of *ARF1*, *COI1*, and *SGT1* enhanced *GmSRC7*-mediated resistance to SMV and TMV. Suppression of *PBS1* expression, however, had no significant effect on *GmSRC7*-mediated TMV resistance. We then validated the roles of *ARF1*, *COI1*, *SGT1*, and *PBS1* in *GmSRC7*-mediated resistance using a transient overexpression system. In the absence of *GmSRC7* expression, overexpression of *ARF1*, *SGT1*, *COI1*, or *PBS1* did not alter viral accumulation. When GmSRC7 was expressed, overexpression of *ARF1*, *SGT1*, or *COI1* reduced *GmSRC7*-mediated resistance to both SMV and TMV. Notably, co-overexpression of *PBS1* and *GmSRC7* did not affect *GmSRC7*-mediated TMV resistance but did reduce *GmSRC7* resistance to SMV (Figure 6).

### 2.4. The Effect of Combined Infection of Candidate Genes on GmSRC7 Resistance

**Figure 7:**
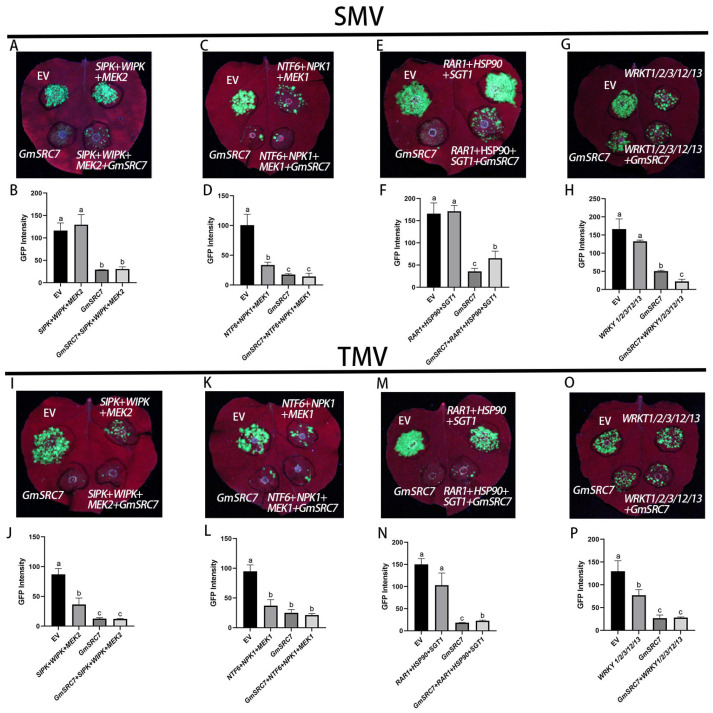
Relationships between signaling pathways and *GmSRC7*-mediated resistance. (**A**,**B**) *MEK2*–*SIPK*–*WIPK* cascade: association with *GmSRC7*-mediated resistance, fluorescence phenotype, and fluorescence intensity during co-expression with SMV. (**C**,**D**) *MEK2*–*SIPK*–*WIPK* cascade: association with *GmSRC7*-mediated resistance, fluorescence phenotype, and fluorescence intensity during co-expression with TMV. (**E**,**F**) *NPK1*–*MEK1*–*NTF6* cascade: association with *GmSRC7*-mediated resistance, fluorescence phenotype, and fluorescence intensity during co-expression with SMV. (**G**,**H**) *NPK1*–*MEK1*–*NTF6* cascade: association with *GmSRC7*-mediated resistance, fluorescence phenotype, and fluorescence intensity during co-expression with TMV. (**I**,**J**) *RAR1*–*HSP90*–*SGT1* cascade: association with *GmSRC7*-mediated resistance, fluorescence phenotype, and fluorescence intensity during co-expression with SMV. (**K**,**L**) *RAR1*–*HSP90*–*SGT1* cascade: association with *GmSRC7*-mediated resistance, fluorescence phenotype, and fluorescence intensity during co-expression with TMV. (**M**,**N**) *WRKY1*/*2*/*3*/*12*/*13* cascade: association with *GmSRC7*-mediated resistance, fluorescence phenotype, and fluorescence intensity during co-expression with SMV. (**O**,**P**) *WRKY1*/*2*/*3*/*12*/*13* cascade: association with *GmSRC7*-mediated resistance, fluorescence phenotype, and fluorescence intensity during co-expression with TMV. The *t*-test was performed between samples in different treatment groups. The same letter indicates no significant difference between groups, while different letters indicate significant differences.

Overexpression of *MEK2*/*WIPK*/*SIPK* did not significantly affect SMV infection but produced a pronounced reduction in TMV infection (Figure 7A,B,I,J). When *GmSRC7* was coexpressed, *MEK2*/*WIPK*/*SIPK* overexpression had no significant effect on either SMV or TMV accumulation.

Overexpression of *NPK1*/*MEK1*/*NTF6* markedly decreased the fluorescence intensity of SMV and TMV, indicating substantial inhibition of their accumulation. When *GmSRC7* was coexpressed, however, *NPK1*/*MEK1*/*NTF6* overexpression no longer produced a significant effect on SMV or TMV accumulation (Figure 7C,D,K,L).

We co-expressed three interacting genes (*RAR1*/*HSP90*/*SGT1*), which regulate R protein stability for disease resistance, together with *GmSRC7* and SMV/TMV in Wt*Nb*. Overexpression of *RAR1*/*HSP90*/*SGT1* alone produced no significant change in SMV or TMV infections (Figure 7E,F,M,N). This result indicates that, in the absence of *GmSRC7*, overexpressing *RAR1*/*HSP90*/*SGT1* does not alter SMV or TMV accumulation and may instead destabilize the R-protein complex, reducing effective resistance. When *GmSRC7* was co-expressed, however, viral titers rose significantly after *RAR1*/*HSP90*/*SGT1* overexpression (Figure 7E,F,M,N), indicating that overexpression of these chaperone components markedly increases SMV and TMV accumulation in the presence of *GmSRC7*.

Five *WRKY* genes (*WRKY1*, *WRKY2*, *WRKY3*, *WRKY12*, and *WRKY13*) were cloned and coexpressed with *GmSRC7* in Wt*Nb*. Their overexpression did not alter SMV infection levels but significantly reduced TMV infection (Figure 7G,H,O,P). When coexpressed with *GmSRC7*, overexpression of these *WRKY* genes significantly enhanced resistance to SMV and had no significant effect on TMV resistance (Figure 7G,H,O,P).

## 3. Discussion

The interaction between plants and pathogens has emerged as a prominent research topic in recent years. This study primarily examined the immune mechanisms by which *GmSRC7* mediates resistance to SMV and TMV. The analysis focused on members of known R protein signaling and anti-SMV pathways in both *Nicotiana* *benthamiana* and *Glycine max*.

Research has shown that TNL and CNL utilize distinct signaling pathways to initiate ETI responses. Most CNLs, such as RPM1, RPS2, and RPS5, rely on the predicted integrin-like protein NDR1 (Non-race specific Disease Resistance 1) to activate immunity. In contrast, most TNLs, including RPP2, RPP4, RPP5, RPP21, and RPS4, depend on the enzyme-like protein EDS1 (Enhanced Disease Susceptibility 1) [74]. The *GmEDS1* gene has also been implicated in soybean resistance to SMV [40]. Our study suggests that *GmEDS1* is either downstream or functionally related to *GmSRC7*. In the VIGS experiment, suppressing *NbEDS1* expression partially reduced fluorescence intensity compared to the empty vector pTRV2. This indicates that inhibiting *NbEDS1* expression partially enhances resistance to SMV and TMV of *GmSRC7*. This effect may occur because, in *N*. *benthamiana*, *GmSRC7* cannot activate downstream *NbEDS1*, and structure of *GmSRC7* differs from the *N* gene, as *GmSRC7* has an atypical BSP domain, while the *N* gene is a typical TNL-type gene.

The *SGT1* (Suppressor of G2 Allele of Skp1) gene was identified following initial studies. Yeast two-hybrid experiments demonstrated that AtRAR1 interacts with AtSGT1a and AtSGT1b, which are homologous to the yeast SGT1 protein [75]. Subsequent research revealed that RAR1 in *tobacco* is involved in *N*-gene-mediated resistance to TMV [76]. In *A*. t*haliana*, *RAR1* and *NDR1* regulate *RPM1*-mediated resistance responses through either parallel or linear signaling pathways [77]. This finding clarified the role of the HSP90–RAR1–SGT1 complex in *R*-gene-mediated defense responses. Numerous studies have documented the complex’s role in disease resistance mediated by *R* genes, such as *N*-gene-mediated resistance to TMV [78,79,80]. In the context of soybean resistance to SMV, *Rsv1*-mediated SMV resistance requires *GmRAR1* and *GmSGT1* but not *GmHSP90* [81]. Given that the *GmSRC7* gene is homologous to the *N* gene, it is possible that GmHSP90, GmRAR1, and GmSGT1 form a complex to protect the *GmSRC7* protein and enable its protective function.

Inhibiting the expression of *NbHSP90* and *NbRAR1* weakens *GmSRC7*-mediated resistance, likely because these proteins function as molecular chaperones that connect with NbSGT1. This suppression prevents the formation of the necessary complex, thereby hindering *GmSRC7*-mediated resistance. Interestingly, silencing the *NbSGT1* gene actually enhances *GmSRC7* resistance, possibly due to the interaction between COI1, SGT1, and the E3 ubiquitin ligase SCF (Skip/Cullin/F-box). Ubiquitin-mediated protein degradation is prevalent in higher organisms and plays a crucial role in plant defense against biotic and abiotic stresses by regulating the biosynthesis and signaling pathways of defense-related hormones and components [64,82]. The antiviral activity of *GmSRC7* may be affected by these ubiquitin-mediated degradation processes. In transient transformation experiments, combinatorial expression results indicated that overexpressing *NbRAR1*/*NbHSP90*/*NbSGT1* significantly enhanced the spread of SMV and TMV when *GmSRC7* was expressed. This may occur because the ubiquitination function outweighs the protective role of molecular chaperones. In soybean transient transformation experiments, silencing *GmSGT1-1* and *GmSGT1-2* significantly reduced resistance to SMV of *GmSRC7*, while silencing *GmHSP90* and *GmRAR1* had no effect, likely due to the dominance of ubiquitination.

ARF1, a positive regulator of the HR response, is part of the Ras superfamily of GTP-binding proteins, also known as GTPases [83]. The gene *NbARF1* plays a crucial role in the immune pathway. AtPBS1 is cleaved by AvrPphB, a cysteine protease effector from *Pseudomonas syringae*. This cleavage causes conformational changes in AtPBS1, allowing it to bind to the LRR domain of RPS5, a member of the NLR protein family, which activates RPS5 and triggers pathogen resistance [84]. Experimental observations indicate that NbPBS1 exhibits antagonistic activity against the SMV resistance of *GmSRC7*. This antagonism likely arises because NbPBS1’s activity is inhibited when it interacts with *GmSRC7*, preventing its binding to RPS5 and thereby weakening SMV resistance in *GmSRC7*-Ox-*Nb* plants.

Discovered in 1998, JAR1 is a protein influencing bacterial resistance in *Arabidopsis* and is part of the jasmonic-acid-related proteins, which inhibit plant seed germination, affect growth, promote aging, and enhance resistance [29]. Our study shows that silencing *GmJAR1* significantly reduces resistance to SMV of *GmSRC7*, highlighting *GmJAR1*’*s* crucial role in the soybean *GmSRC7*-mediated immune response. *GmSGS3*, a key player in plant endogenous gene silencing [39], also shows reduced resistance to SMV when silenced. Overexpression of *SGS3*, a vital component of RNA silencing, has been reported to inhibit AGO1’s siRNA and partially restore the inhibited AGO1 protein, thereby alleviating SMV infection symptoms [39]. This suggests that the *GmSRC7* gene may regulate the RNA interference pathway involving *GmSGS3* to enhance its inhibitory effect on SMV. Although *GmAKT2*, *GmPP2C*, *GmMPK6*, *GmMPK4c*, *GmHSP40.1*, *GmHSP90*, *GmCNX1*, *GmeEF1a*, *GmAGO1*, *GmRAR1*, and *GmPAD4* have been reported to confer resistance to SMV, our findings indicate they are not involved in *GmSRC7*-mediated resistance. Additionally, other resistance genes and loci may regulate certain genes to develop resistance to SMV in soybeans.

Experiments have shown that 14 genes—*NbNRG1*, *NbHSP90*, *NbPAD4*, *NbMEK1*, *NbMEK2*, *NbSIPK*, *NbWIPK*, *NbNTF6*, *NbPR1a*, *NbWRKY1*, *NbWRKY2*, *NbWRKY3*, *NbRAR1*, and *NbNPR1*—work synergistically with *GmSRC7* resistance. Inhibiting these genes’ expression can reduce resistance of *GmSRC7*, likely because they are involved in a disease resistance signaling pathway that aligns with the one mediated by *GmSRC7*.

Experiments with transient overexpression systems in Wt*Nb* and *NahG*-Ox-*Nb* indicate that SA is crucial for *GmSRC7* to develop resistance to SMV. SA plays a significant role in mediating the HR, and resistance to SMV of *GmSRC7* is accompanied by this HR phenotype, suggesting that *GmSRC7* leverages SA for resistance. While SA is recognized as a plant defense response inducer, its precise role in enhancing pathogen resistance remains unclear [85]. Transcriptome data reveal that SMV infection upregulates SA-synthesis-related genes *PAL1* and *ICS1* early in *N*. *benthamiana* infection, with these genes being the sole sources of SA synthesis in plants [71]. Consequently, SA accumulation is vital in the anti-SMV process, leading us to speculate that SA is a key hormone for soybean resistance to SMV. Our study demonstrates that resistance to SMV can be induced through exogenous applications of INA/MeSA in Wt*Nb* and INA in *NahG-*Ox*-Nb*, further underscoring SA’s essential role in combating SMV. However, INA and MeSA differ in their mechanisms; INA is an SA analog, while MeSA requires conversion to SA to be effective. Overexpression of *NahG* inhibits MeSA’s action but not INA’s, whereas overexpression of *GmNPR1*, an SA receptor gene, enhances MeSA’s action without affecting INA. SA-induced early genes encode many key regulatory factors essential for plant immunity. Overexpression of immunomodulators like *SARD1*, *WRKY70*, *SOBIR1*, *ALD1*, *ADR1*, and *EDS1*/*PAD4* has been shown to enhance pathogen resistance, indicating their role in SA-induced immunity [86]. Interestingly, SA treatment rapidly upregulates known negative regulators of plant immunity [87]. These SA-induced genes might be downstream of *GmSRC7* or interact with it. Studies agree that PAD4/EDS1 compensates for SA-mediated defense by activating downstream defense-response genes [88]. Thus, it is likely that *GmSRC7* regulates SA accumulation via the *GmEDS1* gene, conferring SMV resistance. Additionally, research shows that PBS3 protects EDS1 from degradation through the Cul3-E3 ligase mediated by NPR3/NPR4 by binding to EDS1 [69]. PBS3’s role as a key gene in the SA synthesis pathway was reported recently [89]. Therefore, *GmSRC7* might also regulate PBS3 to enhance the SA synthesis pathway or protect EDS1 function. Literature indicates that isochorismate is catalyzed by PBS3 to form isochorismoyl-glutamate A, which is then converted into SA by EPS1 [90]. Thus, *GmSRC7* may regulate SA synthesis genes to produce substantial SA for anti-SMV effects, though further research is necessary to confirm this.

In recent years, leveraging plants’ innate immune responses to combat pests and diseases has become a transformative strategy for enhancing crop yields. This method offers greater stability, environmental sustainability, and efficiency compared to traditional chemical pesticides. The *GmSRC7* gene is potentially key to understanding soybean–pathogen interactions, and studying its upstream and downstream disease resistance pathways could lead to the development of next-generation genetically modified soybean varieties.

## 4. Materials and Methods

### 4.1. Whole Genome Sequence Analysis and Gene Screening

Retrieve the sequences of 20 soybean genes involved in SMV resistance from the soybean genome database (Wm82.a2.v1; https://soybase.org/; accessed on 15 December 2020). Additionally, 36 *Nicotiana benthamiana* genes potentially involved in the downstream signaling pathway of *GmSRC7* were identified from the literature and the NCBI database (https://www.ncbi.nlm.nih.gov, NCBI:txid4100; accessed on 10 March 2021). Gene-specific primers were designed targeting the open reading frame (ORF) regions of all candidate genes using Oligo 6 software (v6.62).

### 4.2. Plant Materials, Gene Cloning, Vector Construction

All plants were cultivated in a standard greenhouse under the following conditions: 25 °C, a 16/8-h light/dark photoperiod, and 60% relative humidity. The plant materials used for transient expression assays included 3 to 4-week-old wild-type *N. benthamiana*, *GmSRC7*-overexpressing *N. benthamiana* (*GmSRC7*-Ox-*Nb*), and *NahG* overexpressing *N. benthamiana* (*NahG*-Ox-*Nb*). Soybean cultivars Williams were also used in this study. Total RNA was extracted from 6 to 8-week-old soybean leaves using the Trizol reagent (YESEN, cat: 10606ES60). First-strand cDNA was synthesized from the extracted RNA using the HiScript II 1st Strand cDNA Synthesis Kit (Vazyme, cat: R212). The ORFs of the candidate genes were amplified from the cDNAs of soybean *Williams* and *N. benthamiana*. The PCR products were cloned into the pCE2-TA/Blunt-Zero vector (Vazyme, cat: C601) and verified by Sanger sequencing. For overexpression assays, the ORFs were cloned into the *Sac*I/*Kpn*I sites of the binary vector pCambia1300, driven by the cauliflower mosaic virus (CaMV) 35S promoter. The constructed recombinant vectors were verified by sequencing and subsequently transformed into *Agrobacterium tumefaciens* strain GV3101. For virus-induced gene silencing (VIGS), a 300-bp specific fragment for each target gene was designed using the online tool at https://vigs.solgenomics.net/ (accessed on 9 April 2021). This fragment was cloned into the *EcoR*V site of the entry vector pQBV3 and then recombined into the destination vector pCB2004B. The VIGS vectors were transformed into *Agrobacterium tumefaciens* strain EHA105.

### 4.3. Transient Expression in N. benthamiana and Glycine Max

*Agrobacterium* strains carrying the recombinant vectors were initially cultured on LB solid medium with appropriate antibiotics at 28 °C for 24 h. The bacterial cells were harvested by centrifugation, resuspended in infiltration buffer (10 mM MES, 10 mM MgCl_2_, 200 µM acetosyringone), pH is 5.6–5.8, and adjusted to the desired optical density at 600 nm (OD_600_). For functional validation, *Agrobacterium* suspensions carrying the candidate genes (final OD_600_ = 0.8) were mixed with suspensions carrying viral infectious clones (SMV-GFP or TMV-GFP; final OD_600_ = 0.4). The mixed cultures were incubated at room temperature in the dark for 1–3 h before being infiltrated into the leaves of 3 to 4-week-old *N. benthamiana* plants using a 1 mL needleless syringe. For VIGS assays, *GmSRC7*-overexpressing *N. benthamiana* plants were co-infiltrated with a mixture of *Agrobacterium* carrying pTRV1 and the recombinant pTRV2 vector (OD_600_ = 0.8 each). Control plants were infiltrated with a mixture of pTRV1 and the empty pTRV2 vector. All infiltrated plants were maintained in the greenhouse. Each experiment included three biological replicates, with each replicate consisting of 20–30 technical replicates (individual infiltration sites). GFP fluorescence was detected at 4–5 days post-infiltration (dpi) using a hand-held long-wave (365 nm) UV lamp. The diffusion intensity of SMV and TMV on the leaves was calculated using Gelpro analyzer software (v4.0), with the *Y*-axis representing the average fluorescence intensity per leaf.

### 4.4. Quantification and Statistical Analysis

The fluorescence intensity of SMV-GFP or TMV-GFP in leaves was processed and quantified using ImageJ software (v1.8.0, National Institutes of Health, Bethesda, MD, USA). The data presented for each gene are derived from 10 technical replicates. For virus titer detection, total RNA was isolated from pooled leaf samples (from 10 individual infection sites) at 4 dpi. Subsequent cDNA synthesis was performed as described above. The relative expression level of viral titer at 3 dpi was determined by quantitative RT-PCR (qRT-PCR) using the SYBR Green method (PerfectStart Green qPCR SuperMix, TransGen Biotech, cat: TG-AQ601) on an Applied Biosystems instrument (ViiA 7, Thermo Fisher Scientific, Waltham, MA, USA). Soybean *β*-actin was used as the internal reference gene. The relative viral accumulation was calculated using the 2^–ΔΔCt^ method, with the virus-only infection set to 100%. The control group (EV) was co-infiltrated with an empty vector and the viral infectious clone. All graphs were generated using GraphPad Prism 8 (v8.3, La Jolla, CA, USA) and Excel 2019 (v16.0 Microsoft, Redmond, WA, USA). Data are presented as the mean ± standard deviation (SD). Statistical significance was determined by Student’s *t*-test. Significance levels are denoted as follows: ns, not significant; *: *p* < 0.05; **: *p* < 0.01; ***: *p* < 0.001.

### 4.5. External Application of Reagents

MeSA is prepared as a 1 mM solution and dissolved in 60% ethanol. INA is a salicylic acid analog, solid, purchased by Yuanye Company, also prepared as a 1 mM solution and dissolved in water. When exogenous application is applied to *N. benthamiana* leaves after infection, use a brush dipped in the solution to be added, and then brush one to two times on the parts of the leaves that need to be provided. Apply evenly on both sides. Apply once every 12 h.

## 5. Conclusions

**Figure 8:**
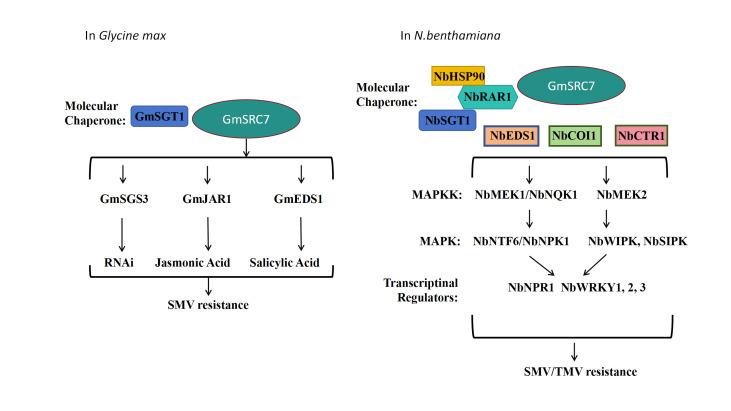
Pathway diagram of *GmSRC7* exerting resistance function.

SA is a key determinant of *GmSRC7*-mediated resistance to SMV. In the absence of SA, resistance to SMV of *GmSRC7* is suppressed. In soybean, *GmSRC7* confers resistance through multiple pathways, including the molecular chaperone pathway (*GmSGT1-1*, *GmSGT1-2*), the lipase hydrolysis pathway (*GmEDS1*), the JA pathway (*GmJAR1*), and the RNA interference pathway (*GmSGS3*) (Figure 8).

VIGS revealed that members of the WRKY transcription factor family, chaperone proteins, the ethylene pathway, the MAPK cascade, the SA and JA pathways, calcium-dependent protein kinases, nuclear migration proteins, and other *R* genes act synergistically in mediating *GmSRC7* resistance to SMV and TMV (Figure 8).

## Data Availability

The data used in this research are publicly available. Each cloned gene can be found at https://www.ncbi.nlm.nih.gov/ (accessed on 20 December 2020). The data (results) presented in this research are available in the Supplementary Materials. Please refer to the literature published by Zhao Q, et al. [91] for the transcriptome data in the Supplementary Data.

## Supplementary Materials

The following supporting information can be downloaded at: https://www.mdpi.com/article/10.3390/plants15020318/s1, Table S1: Basic information of host genes participated in SMV resistance; Table S2: SA related genes; Table S3: Basic information of 37 *N. benthamiana* genes related to disease resistance pathways; Table S4: Primers used in this study; Figure S1: Detect the expression level of the target genes of soybean leaves after interference; Figure S2: Heatmap of differentially expressed SA related genes at different dpi; Figure S3: Phenotype after interference with 6 genes (*AOX1*, *TpoxC1*, *RDR6*, *MYB1*, *NPK1* and *CTR1*); Figure S4: Phenotype after interference with 5 genes (*EDS1*, *COI1*, *PBS1*, *SGT1*, and *ARF1*); Figure S5: Verification of gene expression levels after VIGS; Figure S6: Verification of gene expression level after RNA interference.
