# Supplementary material for: Signaling Pathway Analysis and Downstream Genes Associated with Disease Resistance Mediated by GmSRC7
Source: Plants (Basel). 2026 Jan 21;15(2):318. doi: 10.3390/plants15020318 (PMC12845291; doi:10.3390/plants15020318)
Supplement: Supplementary file 1 [file plants-15-00318-s001.zip › Figure S1.pdf]

Supplement Figure S1

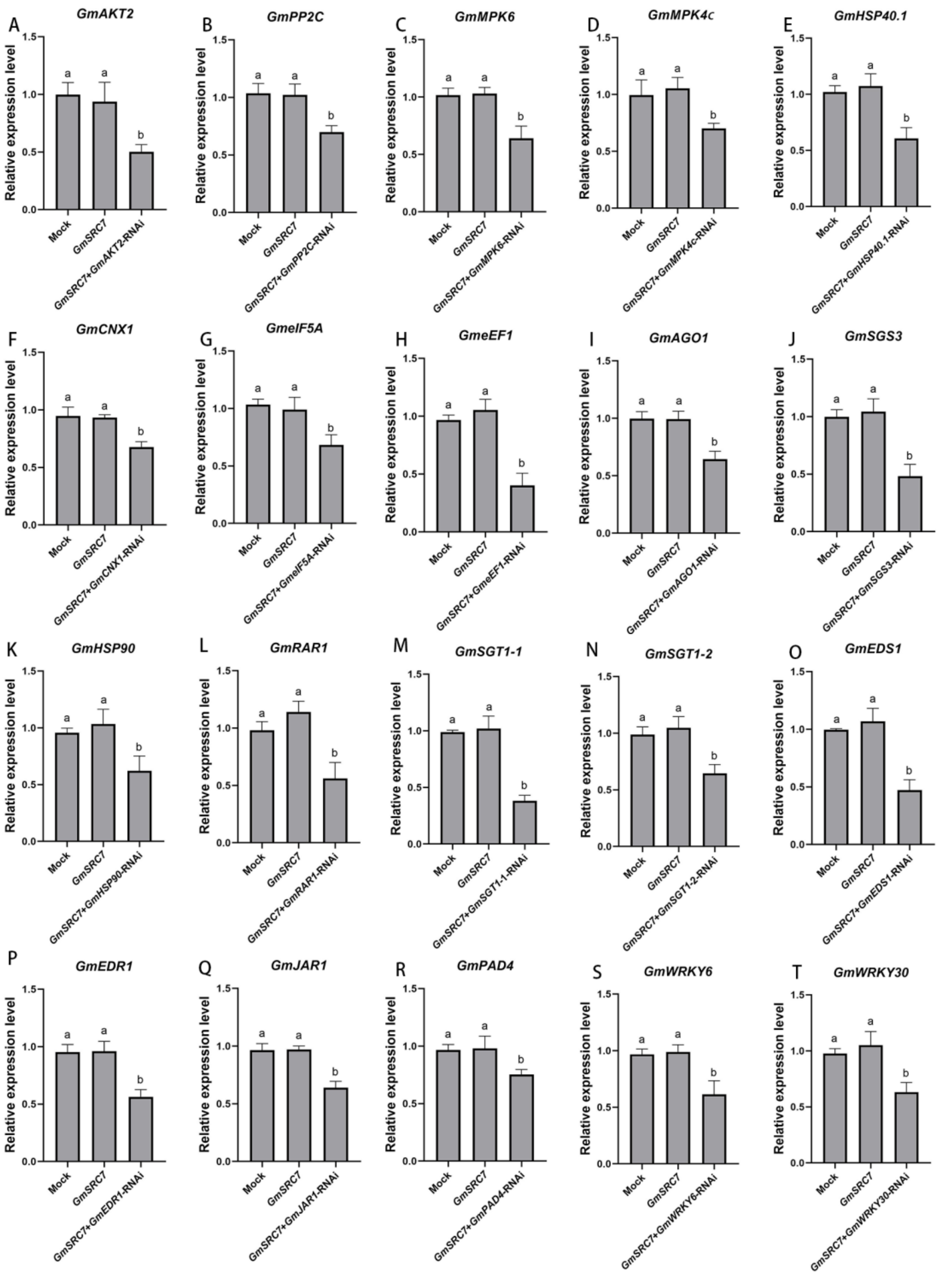

Supplement Figure S1. Expression level of the candidate genes detection after transient Silence in soybean leaves using the leaf injection method. Silencing with (A) *GmAKT2*, (B) *GmPP2C*, (C) *GmMPK6*, (D) *GmMPK4*, (E) *GmHSP40.1*, (F) *GmHSP90*, (G) *GmCNX1*, (H) *GmeIF5A*, (I) *GmeEF1*, (J) *GmAGO1*, (K) *GmSGS3*, (L) *GmRAR1*, (M) *GmSGT1-1*, (N) *GmSGT1-2*, (O) *GmEDS1*, (P) *GmEDR1*, (Q) *GmJAR1*, (R) *GmPAD4*, (S) *GmWRKY6*, and (T) *GmWRKY30*.
