# Supplementary material for: Signaling Pathway Analysis and Downstream Genes Associated with Disease Resistance Mediated by GmSRC7
Source: Plants (Basel). 2026 Jan 21;15(2):318. doi: 10.3390/plants15020318 (PMC12845291; doi:10.3390/plants15020318)
Supplement: Supplementary file 1 [file plants-15-00318-s001.zip › Figure S2.pdf]

Supplement Figure S2

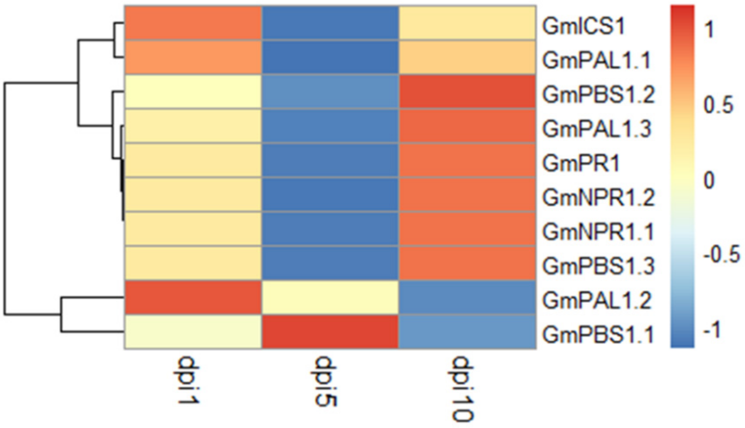

Supplement Figure S2. Heatmap of differentially expressed SA related genes at different dpi

The expression levels of SA related genes showed significant changes when SMV infected soybeans. When SMV infects soybean plants at 1 dpi, *GmICS1*, *GmPAL1.1*, and *GmPAL1.2* are up regulated. When SMV infects soybean plants at 5 dpi, the *GmPBS1.1* gene up regulated, *GmICS1*, *GmPAL1.1*, *GmPBS1.2*, *GmPAL1.3*, *GmPR1*, *GmNPR1.2*, *GmNPR1.1*, *GmPBS1.3* were down regulated. When SMV infects soybean plants at 10 dpi, *GmPBS1.2*, *GmPAL1.3*, *GmPR1*, *GmNPR1.2*, *GmNPR1.1*, and *GmPBS1.3* were up regulated, while *GmPAL1.2* and *GmPBS1.1* were down regulated.
