# Supplementary material for: Signaling Pathway Analysis and Downstream Genes Associated with Disease Resistance Mediated by GmSRC7
Source: Plants (Basel). 2026 Jan 21;15(2):318. doi: 10.3390/plants15020318 (PMC12845291; doi:10.3390/plants15020318)
Supplement: Supplementary file 1 [file plants-15-00318-s001.zip › Figure S3.pdf]

Supplementary Figure S3

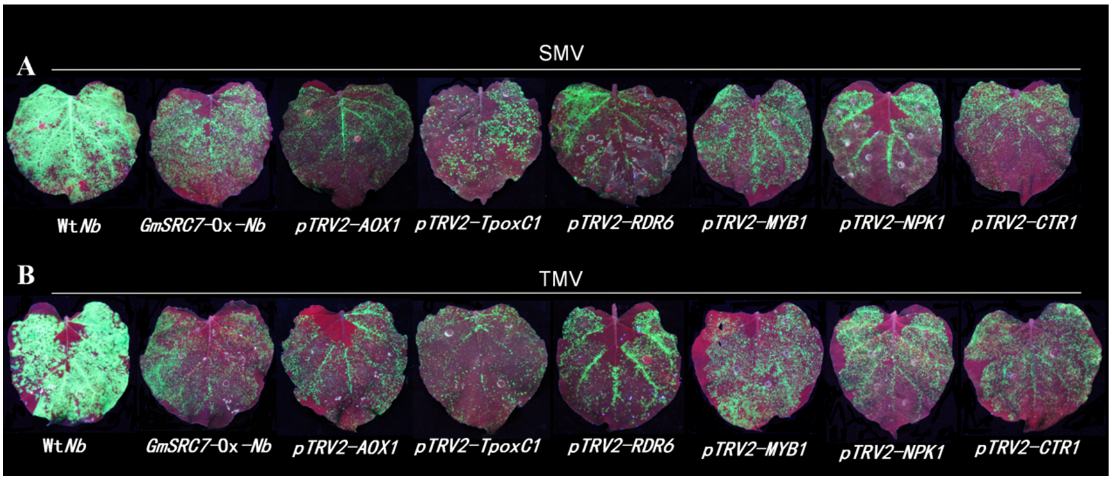

Supplementary Figure S3, VIGS system mediated gene silencing enhances the phenotype of *GmSRC7* resistance. A. The spread of SMV after 5 days of infection; B. The spread of TMV after 5 days of infection. Wt*Nb* is the positive control, *GmSRC7*-Ox-*Nb* represents overexpressing *GmSRC7* transgenic *N.benthamiana* leaves as a negative control, and the rest represent overexpressing *GmSRC7* transgenic *N.benthamiana* experimental leaves.
