# Supplementary material for: Signaling Pathway Analysis and Downstream Genes Associated with Disease Resistance Mediated by GmSRC7
Source: Plants (Basel). 2026 Jan 21;15(2):318. doi: 10.3390/plants15020318 (PMC12845291; doi:10.3390/plants15020318)
Supplement: Supplementary file 1 [file plants-15-00318-s001.zip › Figure S4.pdf]

Supplementary Figure S4

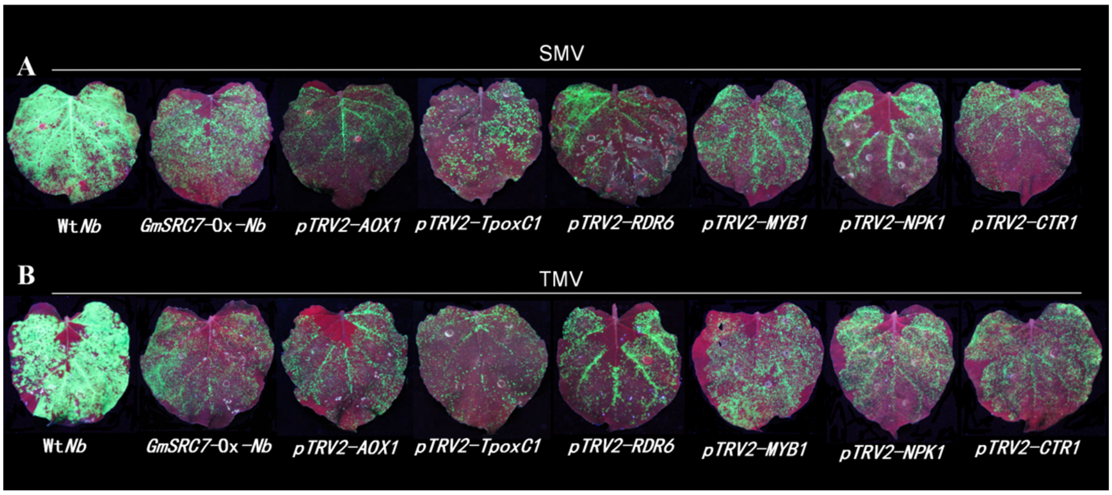

Supplementary Figure S4. The phenotype image of SMV-GFP infection in *GmSRC7*-Ox-*Nb* plants following VIGS is presented. Wt*Nb* served as the positive control, while *GmSRC7*-Ox-*Nb*, representing the overexpressed *GmSRC7* transgenic *Nicotiana benthamiana*, functioned as the negative control. The experimental group employed the *GmSRC7*-Ox-*Nb* plant. A. The spread of SMV after 5 days of infection; B. The spread of TMV after 5 days of infection. Virus accumulation was detected under UV irradiation 5 days post-SMV-GFP infection, following a 10-day period of target gene silencing via VIGS.
