# Supplementary material for: Signaling Pathway Analysis and Downstream Genes Associated with Disease Resistance Mediated by GmSRC7
Source: Plants (Basel). 2026 Jan 21;15(2):318. doi: 10.3390/plants15020318 (PMC12845291; doi:10.3390/plants15020318)
Supplement: Supplementary file 1 [file plants-15-00318-s001.zip › Figure S5.pdf]

Supplementary Figure S5

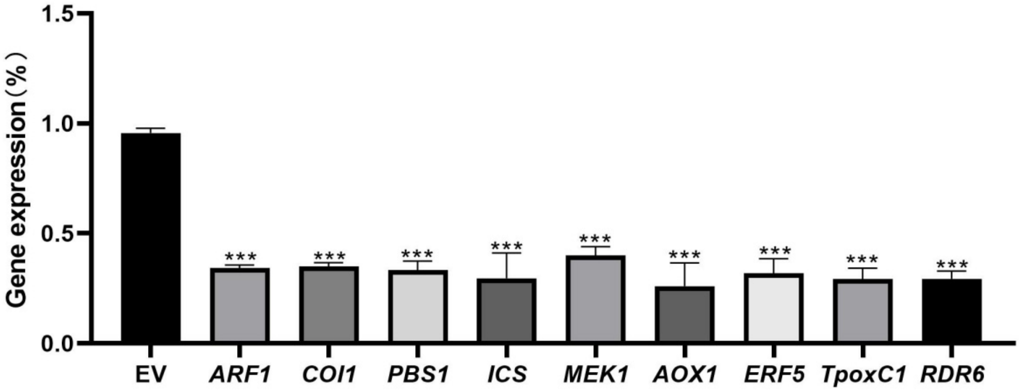


Supplementary Figure S5. Verification of gene expression levels after interference. EV: Control; *ARF1*, *COI1*, *PBS1*, *ICS*, *MEK1*, *AOX1*, *ERF5*, *TpoxC1*, and *RDR6*: Expression levels of *ARF1*, *COI1*, *PBS1*, *ICS*, *MEK1*, *AOX1*, *ERF5*, *TpoxC1*, and *RDR6* in Ox-*GmSRC7* N. benthamiana plants following interference. Target-gene expression was measured by RT-qPCR. Relative to GmSRC7-Ox-*Nb* plants infected with the pTRV2 empty vector, silencing of *ARF1*, *COI1*, *PBS1*, *ICS*, *MEK1*, *AOX1*, *ERF5*, *TpoxC1*, and *RDR6* in GmSRC7-Ox-*Nb* plants resulted in a significant downregulation of the corresponding target-gene expression. Measure the target gene expression after interference by RT-qPCR. Compared with GmSRC7-Ox-*Nb* plants infected with the pTRV2 empty vector, silencing GmSRC7 in transgenic N. benthamiana plants carrying *ARF1*, *COI1*, *PBS1*, *ICS*, *MEK1*, *AOX1*, *ERF5*, *TpoxC1*, or *RDR6* resulted in a significant downregulation of the target gene.
