# Supplementary material for: Signaling Pathway Analysis and Downstream Genes Associated with Disease Resistance Mediated by GmSRC7
Source: Plants (Basel). 2026 Jan 21;15(2):318. doi: 10.3390/plants15020318 (PMC12845291; doi:10.3390/plants15020318)
Supplement: Supplementary file 1 [file plants-15-00318-s001.zip › Figure S6.pdf]

Supplementary Figure S6

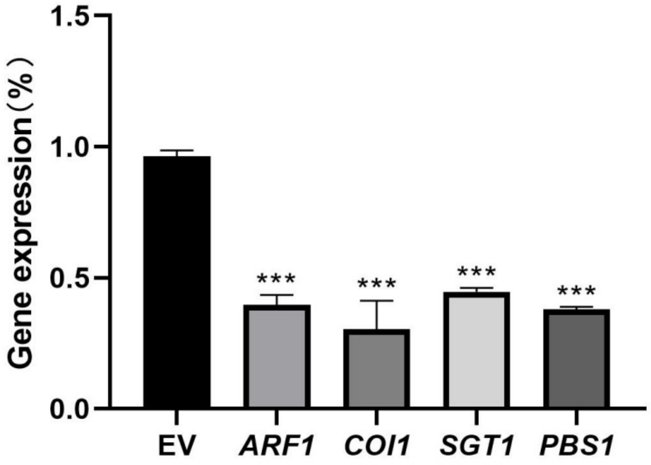

Supplementary Figure S6. Verification of Gene Expression Levels. RT-qPCR validation of the silencing efficiency for *ARF1*, *COI1*, *PBS1*, and *SGT1*. EV: Expression in Ox-GmSRC7 transgenic N. benthamiana plants infected with the pCB2004B empty vector, used as the control. *COI1*, *PBS1*, *ARF1*, and *SGT1*: Expression levels of *ARF1*, *COI1*, *PBS1*, and *SGT1* in Ox-GmSRC7 transgenic N. benthamiana plants following gene interference.
