# Supplementary material for: Signaling Pathway Analysis and Downstream Genes Associated with Disease Resistance Mediated by GmSRC7
Source: Plants (Basel). 2026 Jan 21;15(2):318. doi: 10.3390/plants15020318 (PMC12845291; doi:10.3390/plants15020318)
Supplement: Supplementary file 1 [file plants-15-00318-s001.zip › Table S1.pdf]

Supplement Table S1

Supplement Table S1. Basic information of host genes participated in SMV resistance

| **Gene name** | **Accession number** | **cDNA length** | **Length of interference fragment** | **The protein encoded** | **Brief description of functions** |
|---|---|---|---|---|---|
| *GmAKT2* | Glyma08g20030 | 1785bp | 361bp | $K^+$ transport protein | Regulate $K^+$ in the phloem Transportation |
| *GmPP2C* | Glyma14g32430 | 1160bp | 471bp | Protein phosphatase | Regulate the ABA signaling network |
| *GmMPK6* | Glyma02g15690 | 1176bp | 324bp | Signal transducer and activator of protein kinase | Activate their cellular localization and interactions with transcription factors and changes in phosphorylation |
| *GmMPK4* | Glyma09g39190 | 1122bp | 305bp | Signal transducer and activator of protein kinase | Activation of subcellular localization and changes in interactions with transcription factors and phosphorylation |
| *GmHSP40.1* | Glyma15g057800 | 1494bp | 347bp | Heatshock protein | Catalyzes the formation of disulfide bonds |
| *GmCNX1* | Glyma02g272700 | 1959bp | 467bp | Nitrate reductase | Participate in the biosynthesis of plant Mo |
| *GmeIF5A* | Glyma01g054000 | 354bp | 310bp | Eukaryotic translation initiation factor | Participates in Rsv1-mediated LSHR signaling pathway |
| *GmeEF1* | Glyma17g186600 | 1344bp | 422bp | Elongation factor | Participate in the replication of SMV in soybean |
| *GmAGO1* | Glyma16g34300 | 3162bp | 344bp | AGO protein | Translation inhibition or cleavage of complementary target mRNA in the miRNA pathway |
| *GmSGS3* | Glyma05g198500 | 2063bp | 487bp | Silent inhibitors | An important component of RNAsilence |
| *GmHSP90* | Glyma18g074100 | 2109bp | 499bp | Heatshock protein | It mediates resistance to various pathogens through structurally diversified R protein recruitment |
| *GmRAR1* | Glyma10g38720 | 672bp | 411bp | Mla12-mediated resistance required protein | Participate in R gene-mediated disease resistance |
| *GmSGT1-1* | Glyma01g43150 | 1083bp | 407bp | The promoter of the G2 allele of skp1 | Regulates R protein-mediated defense signal transduction |
| *GmSGT1-2* | Glyma09g23980 | 1080bp | 403bp | The promoter of the G2 allele of skp1 | Regulates R protein-mediated defense signal transduction |
| *GmEDS1* | Glyma06g19920 | 1854bp | 416bp | Eukaryotic lipase | Participate in the defense response mediated by SA and R genes |
| *GmEDR1* | Glyma10g30070 | 2793bp | 366bp | Raf-like MAPKK kinase | Regulate plant disease resistance immunity and MAPK mediated disease resistance response |
| *GmJAR1* | Glyma07g06370 | 1749bp | 424bp | Molassesic acid amide synthase | Regulate the jasmone pathway |
| *GmPAD4* | Glyma13g04540 | 1749bp | 464bp | Lipase | The upstream SA pathway plays a role in promoting plant resistance |
| *GmWRKY6* | Glyma07g39250 | 1731bp | 443bp | Transcription factor | Related to plant senescence and disease resistance |
| *GmWRKY30* | Glyma03g33380 | 1089bp | 457bp | Transcription factor | Promote plant resistance |
