# Supplementary material for: Signaling Pathway Analysis and Downstream Genes Associated with Disease Resistance Mediated by GmSRC7
Source: Plants (Basel). 2026 Jan 21;15(2):318. doi: 10.3390/plants15020318 (PMC12845291; doi:10.3390/plants15020318)
Supplement: Supplementary file 1 [file plants-15-00318-s001.zip › Table S2.pdf]

Supplement Table S2

Supplement Table S2. SA related genes

**Gene name**	**Accession number**	**cDNA length**	**The protein encoded**	**functions**
AtS3H	BT010537	1050bp	Salicylic acid 3-hydroxylase	Degradation of Salicylic Acid
AtS5H	NM_122361	1026bp	Salicylic acid 5-hydroxylase	Degradation of Salicylic Acid
NahG	YP_534831	1305bp	salicylate hydroxylase	Degradation of Salicylic Acid
GmNPR1	NC_038245.1	1773bp	SA receptor	Participate in SA mediated disease resistance pathways
