# Supplementary material for: Signaling Pathway Analysis and Downstream Genes Associated with Disease Resistance Mediated by GmSRC7
Source: Plants (Basel). 2026 Jan 21;15(2):318. doi: 10.3390/plants15020318 (PMC12845291; doi:10.3390/plants15020318)
Supplement: Supplementary file 1 [file plants-15-00318-s001.zip › Table S3.pdf]

Supplement Table S3

Supplement Table S3. Basic information of 36 *Nb* genes related to disease resistance pathways

Gene name	Accession number	cDNA length	The protein that encodes it	Brief description of functions
NbNRG1	DQ054580.1	2553bp	R protein	Located downstream of EDS1, it is a key link in the defense signal transduction mediated by various disease resistance gene-regulated R proteins
NbARF1	DQ531849.1	546 bp	ADP-ribosylating factor	It plays many physiological roles in living organisms, such as gene expression, cytoskeleton reorganization, microtubule formation, vesicle and nuclear pore transport
NbEDS1	AF479625.1	1 824 bp	R protein	Important defense genes are the key nodes of plant immune signaling network
NbHSP90	AY368904.1	2100 bp	Heatshock protein	Molecular partners interact with SGT1 and RAR1
Nb HSP 20	Niben101Scf02290g07003.1	681 bp	Heatshock protein	Participate in regulating protein folding and gene expression
NbSIPK	AB373025.1	1182 bp	Salicylate-induced kinase	Regulated by MAPK pathway and induced by SA, it can be activated by a variety of pathogenic signals and injuries, and is an important component of many signal pathways
NbWIPK	AB098729.1	1130 bp	Wound-induced protein kinase	Interacts with SIPK to exert disease resistance
NbSGT1	AF494083.1	1 113 bp	The promoter of the G2 allele of Skp1	To function in R gene-mediated resistance, it may require SCF-mediated ubiquitination by coupling HSP90 to the SCF complex, which ubiquitinates downstream HSP90 proteins
NbAOX1	KF367455.1	1059 bp	Mitochondrial alternative oxidase	Participate in plant resistance response
NbMEK1	AB360635.1	1065 bp	Cellular extracellular signal-regulated kinase activated by mitogen	Participates in the activation of the M A PK cascade
NbMEK2	AB360636.1	1119 bp	Cellular extracellular signal-regulated kinase activated by mitogen	Participates in MAPK cascade pathway
NbNTF6	AB360634.1	1116 bp	Tobacco Fus-3-like kinase	It participates in MAPK cascade pathway, plays a role in N gene-mediated resistance of tobacco mosaic virus, and has upstream and downstream activation relationship with NPK1 and MEK1
NbRdRp1m	AY574374.1	1440 bp	RNA-dependent RNA polymerase	Induced by SA and involved in the TMV resistance pathway
Nb RDR6	AY722008.1	3594 bp	RNA-dependent RNA polymerase	Participated in the antiviral RNA silencing pathway stimulated by temperature increase but inhibited by the virus-encoded silencing factor
NbERF3	AB573717.1	684 bp	Ethylene response factor	It plays a role in SIPK/WIPK and downstream of WRKY1
NbERF5	Niben101Scf07310g01001.1	672 bp	Ethylene response factor	Responding to different pathogens

Gene name	Accession number	cDNA length	The protein that encodes it	Brief description of functions
				By coordinating chitin and other defense pathways in plants
NbPBS1	MK140809.1	1347 bp	Protein kinase superfamily protein	The RPS5 protein is involved in signal transduction to trigger the plant innate immune response (ETI)
NbICS	LC222288.1	1773 bp	Isochorismate synthase	Participate in salicylic acid synthesis
NbRAR1	LC314308.1	666 bp	MLa 12 protein required for resistance	Regulate the R gene disease resistance signaling pathway and activate the SCF and COP9 complexes
NbWRKY1	Niben101Scf07682g00001.1	714bp	Transcription factor	It is related to plant cell apoptosis and disease resistance
NbWRKY2	Niben101Scf00574g02005.1	960bp	Transcription factor	Participate in plant resistance processes
NbWRKY3	Niben101Scf03739g05005.1	750bp	Transcription factor	Plant growth and development and disease resistance
NbWRKY12	AB711133.1	1578bp	Transcription factor	Affect plant growth, development, flowering and disease resistance
NbWRKY13	AB711134.1	1290bp	Transcription factor	Affect plant growth, development, flowering and disease resistance
NbMYB1	Niben101Scf03570g04002.1	825bp	MYB transcription factor	Activating transcription factor
NbNPK1	Niben101Scf00081g00010.1	2067bp	Tobacco protein kinase	It participates in MAPK cascade pathway and plays a role in N gene-mediated resistance of tobacco mosaic virus
NbNPR1	Niben101Scf14780g01001.1	1767bp	Non-expressing genes of PR	The disease resistance is exerted through the SA pathway
Nb CDPK 2	AJ344156.1	1746bp	Calcium-dependent protein kinase	It is involved in various physiological processes such as resistance to biological and abiotic stress, hormone signaling and development
NbCOI1	Niben101Scf02280g08005.1	942bp	Toxin insensitive protein	JA receptor protein binds to JA activated form to form a transcriptional repressor
NbCTR1	Niben101Scf17760g00008.1	780bp	This is a triple reaction protein	It has a negative control effect on ethylene
NbPR1a	Niben101Scf00107g03008.1	507bP	Pathogenesis related proteins	Participate in salicylic acid disease resistance
NbEREBP1	Niben101Scf00454g04003.1	711bp	Vinyl reactive element binding protein	Plasmid elements that initiate ethylene response during resistance
NbPAD4	Niben101Scf02544g01012.1	1491bp	Lipase	Upstream genes of salicylic acid signaling pathway
NbTpoxC1	Niben101Scf23113g01008.1	969bp	Tobacco peroxidase	Participates in the N gene-mediated resistance pathway
NbCYBP	Niben101Scf01259g04002.1	747bp	Calcium cyclin-binding protein	It may participate in calcium-dependent ubiquitination pathway and protein degradation; it may be a key component of ubiquitin E3 complex.
NbNUDC	Niben101Scf02839g03013.1	909bp	Nuclear transporters	Participates in controlling cell migration
