# Supplementary material for: Signaling Pathway Analysis and Downstream Genes Associated with Disease Resistance Mediated by GmSRC7
Source: Plants (Basel). 2026 Jan 21;15(2):318. doi: 10.3390/plants15020318 (PMC12845291; doi:10.3390/plants15020318)
Supplement: Supplementary file 1 [file plants-15-00318-s001.zip › Table S4.pdf]

Supplement Table S4

Supplement Table S4. The primers used in this study

**Primer Name**	**Sequence (5′-3′)**
GmAKT2-ORF-F	GGATCTTCCAGAGATGGATCCATGCTAGCTGATCGTTACCC
GmAKT2-ORF-R	CTGCCGTTCGACGATCCCGGGTCAGCTTATCCAACAAAAAA
GmPP2C-ORF-F	GGATCTTCCAGAGATGGATCCATGTTCAGTTCGTTGCATGC
GmPP2C-ORF-R	CTGCCGTTCGACGATCCCGGGTCAATCAGTTGTTACTGATC
GmHSP40.1-ORF-F	GGATCTTCCAGAGATGGATCCATGGATGGTCACGGAGGAGG
GmHSP40.1-ORF-R	CTGCCGTTCGACGATCCCGGGGTAATGACCCTTAACCTTAT
GmMPK6-ORF-F	GGATCTTCCAGAGATGGATCCATGGAAGGAGGAGGAGCTGC
GmMKP6-ORF-R	CTGCCGTTCGACGATCCCGGGCTACTGCTGATACTCAGGGT
GmMPK4-ORF-F	GGATCTTCCAGAGATGGATCCATGTCTGCTGTTGAGTCAGC
GmMPK4-ORF-R	CTGCCGTTCGACGATCCCGGGTCAGTAGACTGGTGGAACAG
GmSGT1-1-ORF-F	GGATCTTCCAGAGATGGATCCATGGCTACGGCGCTGGAGAA
GmSGT1-1-ORF-R	CTGCCGTTCGACGATCCCGGGTCAATACTCCCATTTTTTCA
GmSGT1-2-ORF-F	GGATCTTCCAGAGATGGATCCATGGCTTCCGATCTCGAAGC
GmSGT1-2-ORF-R	CTGCCGTTCGACGATCCCGGGTTAATATTCCCATTTCTTCA
GmEDR1-ORF-F	GGATCTTCCAGAGATGGATCCATGAGTGAGAGAGTGAAGAA
GmEDR1-ORF-R	CTGCCGTTCGACGATCCCGGGTCAGGGGGTGGAATTTACAG
GmPAD4-ORF-F	GGATCTTCCAGAGATGGATCCATGACCCTTCCTCAATCATT
GmPAD4-ORF-R	CTGCCGTTCGACGATCCCGGGCTAAGCCTGAGGGGGGAAAT
GmWRKY30-ORF-F	GGATCTTCCAGAGATGGATCCATGGAAGAGAGCACCAAGAG
GmWRKY30-ORF-R	CTGCCGTTCGACGATCCCGGGTTATGAAGAGAAAAATTCAG
GmCNX1-ORF-F	GGATCTTCCAGAGATGGATCCATGATCTCCGTCATTGACGC
GmCNX1-ORF-R	CTGCCGTTCGACGATCCCGGGTTACGTGCAACAGGAACAAG
GmeIF5A-ORF-R	GGATCTTCCAGAGATGGATCCATGTCAGACGAGGAACACCA
GmeIF5A-ORF-F	CTGCCGTTCGACGATCCCGGGTTAGAAGAAAAGTGTAATGA
GmAGO1-ORF-R	GGATCTTCCAGAGATGGATCCATGGTCAGAAAGAGAAGAAC
GmAGO1-ORF-F	CTGCCGTTCGACGATCCCGGGTTAACAATAAAACATAACTC

GmSGS3-ORF-F	GGATCTTCCAGAGATGGATCCATGCCAAGTCAACTTAAAGT
GmSGS3-ORF-R	CTGCCGTTCGACGATCCCGGGTCAGATACCATTGGACTTTT
GmRAR1-ORF-F	GGATCTTCCAGAGATGAGCTCATGGAGAAGACAGCAGTTAA
GmRAR1-ORF-R	CTGCCGTTCGACGATCGGCCGTCAGGACTCTGGATCCGCAT
GmHSP90-ORF-F	GGATCTTCCAGAGATGAGCTCATGGCGGAGACCGAGACGTT
GmHSP90-ORF-R	CTGCCGTTCGACGATCGGCCGTTAATCAACCTCCTCCATTT
GmEDS1-ORF-F	GGATCTTCCAGAGATGAGCTCATGAAGAATTATAGAATTAG
GmEDS1-ORF-R	CTGCCGTTCGACGATCGGCCGCTAGGAAGAAGTGTCCTTGA
GmJAR1-ORF-F	GGATCTTCCAGAGATGAGCTCATGTTGGAGAAAGTTGAAGA
GmJAR1-ORF-R	CTGCCGTTCGACGATCGGCCGTCAATTGAAGGCAGTACTGA
GmWRKY6-ORF-F	GGATCTTCCAGAGATGAGCTCATGAATAATCCATTTGGTCT
GmWRKY6-ORF-R	CTGCCGTTCGACGATCGGCCGCTATTGATTAATTGTAGCAC
GmeEF1A-ORF-F	GGATCTTCCAGAGATGAGCTCATGGGTAAGGAAAAGGTTCA
GmeEF1A-ORF-R	CTGCCGTTCGACGATCGGCCGTCACTTCTTCTTCTGGGCAG
GmeNPR1-ORF-F	GAGAACACGGGGGACGAGCTCAGGGAGTTTTGAAGTATGAA
GmeNPR1-ORF-R	ATTTGTTGGATCCCGGGTACCGGGAGATTAAAGGTACATCT
ATEPS1-ORF-F	GAGAACACGGGGGACGAGCTCATGGAAGAAGAATTGGTGGT
ATEPS1-ORF-R	ATTTGTTGGATCCCGGGTACCTCATACAATGCTTACATATT
ATS3H-ORF-F	GAGAACACGGGGGACGAGCTCATGGAAGAAGAATTGGTGGT
ATS3H-ORF-R	ATTTGTTGGATCCCGGGTACCTTAGGTTGTTGGAGCTTTGA
ATS5H-ORF-F	GAGAACACGGGGGACGAGCTCATGGAAGAAGAATTGGTGGT
ATS5H-ORF-R	ATTTGTTGGATCCCGGGTACCTTAGTTGTTTAGAAAATTCT
ATPBS3-ORF-F	GAGAACACGGGGGACGAGCTCATGGAAGAAGAATTGGTGGT
ATPBS3-ORF-R	ATTTGTTGGATCCCGGGTACCTCAAATACTGAAGAATTTGG
GmAKT2-RNAi-F	GGCTCAGGGGATATCATGAAGCACCAGATGATGT
GmAKT2-RNAi -R	GTGCAGGGCGATATCGCCACAGTTAGCAAGTTC
GmPP2C-RNAi-F	GGCTCAGGGGATATCGAGCAGGAAGGAGATGGA
GmPP2C-RNAi-R	GTGCAGGGCGATATCTCGTGAAGTGGCAAGAAC
GmHSP40.1-RNAi-F	GGCTCAGGGGATATCACGAGTATCCGAAGGTGTA

Gm HSP40.1-RNAi-R	GTGCAGGGCGATATCACGCCAGTCATCATCATC
GmMPK6-RNAi-F	GGCTCAGGGGATATCATGTATGGTCTGTTGGTTGT
GmMKP6-RNAi-R	GTGCAGGGCGATATCGCACACTGGTTCATCACT
GmMPK4-RNAi-F	GGCTCAGGGGATATCATAGGTTCGCCAGATGATG
GmMPK4-RNAi-R	GTGCAGGGCGATATCTCCAGATGAGTTCCTTGATG
GmSGT1-1-RNAi-F	GGCTCAGGGGATATCACTACCAGAAGCCAGAAGA
GmSGT1-1-RNAi-R	GTGCAGGGCGATATCAGCAGCATCACCATCAAG
GmSGT1-2-RNAi-F	GGCTCAGGGGATATCTTACTGTTGACTTCGGTGAA
GmSGT1-2-RNAi-R	GTGCAGGGCGATATCGCTCTTCTTGTATCCTCATCT
GmEDR1-RNAi-F	GGCTCAGGGGATATCGGACTCTTGGAATGAATACAG
GmEDR1-RNAi-R	GTGCAGGGCGATATCACGGCTTGGAACTACTAAG
GmPAD4-RNAi-F	GGCTCAGGGGATATCAGCAACAACACCTGAACAT
GmPAD4-RNAi-R	GTGCAGGGCGATATCATCCTCTTCCAGCCACTC
GmWRKY30-RNAi-F	GGCTCAGGGGATATCTCAATCAACCACAAGACAGT
GmWRKY30-RNAi-R	GTGCAGGGCGATATCCTTCCTCCACCAGCATTAG
GmCNX1-RNAi-F	GGCTCAGGGGATATCCGGCTTCGTCAACCAATA
GmCNX1-ORF-R	GTGCAGGGCGATATCACAACTCTTGCTCCTCCTA
GmeIF5A-RNAi-R	GGCTCAGGGGATATCAGACGAGGAACACCACTT
GmeIF5A-RNAi-F	GTGCAGGGCGATATCCTGAAATCTTACAAACCCATCC
GmAGO1-RNAi-R	GGCTCAGGGGATATCGAACCTGCTCCTACTCCT
GmAGO1-RNAi-F	GTGCAGGGCGATATCGCTCCTTCATCATCATCAAC
GmSGS3-RNAi-F	GGCTCAGGGGATATCCACTATGGCAGACATTGGT
GmSGS3-RNAi-R	GTGCAGGGCGATATCCAGGCTATCATCAACACTCT
GmRAR1-RNAi-F	GGCTCAGGGGATATCTACACCAGTGAAGAAGAACA
GmRAR1-RNAi-R	GTGCAGGGCGATATCCACGGAGGAATGCTCATAA
GmHSP90-RNAi-F	GGCTCAGGGGATATCGCAGAGAACAAGGAGGATT
GmHSP90-RNAi-R	GTGCAGGGCGATATCCAACAACACGGTCAGAGA
GmEDS1-RNAi-F	GGCTCAGGGGATATCATGACATAGTGCCAAGGATT
GmEDS1-RNAi-R	GTGCAGGGCGATATCATAGTTCAGGTGTTGCGATA

GmJAR1-RNAi-F	GGCTCAGGGGATATCCACGGAGAGGATGATGGA
GmJAR1-RNAi-R	GTGCAGGGCGATATCCTGCTTGCTGCTGTAGATA
GmWRKY6-RNAi-F	GGCTCAGGGGATATCGATTCCTCTCACTCTTCCTC
GmWRKY6-RNAi-R	GTGCAGGGCGATATCCGTTGGTTATGTTGCTGTT
GmeEF1A-RNAi-F	GGCTCAGGGGATATCGCTGACTGTGCTGTTCTT
GmeEF1A-RNAi-R	GTGCAGGGCGATATCGTTCCAATACCTCCAATCTTG
Gm- β-actin-qPCR-F	CCACCAACATATCGTCATCA
Gm- β-actin-qPCR-R	ACAAATTCTGAGGGACTAGC
GFP-qPCR-F	CCTGTTCCATGGCCAACACT
GFP-qPCR-R	CAGCACGCGTCTTGTAGTTC
***NbNRG*1-ORF-1F**	ATGGCCGCAACTCTGTTGGGAGGAG
***NbNRG1*-ORF-2F**	TCCCAAACAAGTTGCGGGTCGTTAAGTAAAAC
***NbNRG1*-ORF-R**	GTCCCAAAGTCGCCTCAAGAAACTTCTCT
***NbARF1*-ORF-F**	ATGGGGCTAACATTCACCAAGCTTTT
***NbARF1*-ORF-R**	CTAGGACTTGTTGGCAATGTTGTTAG
***NbEDS1*-ORF-F**	ATGGTGAGAATTGAAGAGGGAGAGAAGTGAA
***NbEDS1*-ORF-R**	CTAAGAATTTACTTTCCCT
***NbHSP90*-ORF-F**	ATGGCGGAGGCAGAGACGTTTGCA
***NbHSP90*-ORF-R**	TTAGTCAACTTCCTCCATCTTGCTACCTTCAG
***NbSIPK*-ORF-F**	ATGGATGGTTCTGGTCAGCAGACG
***NbSIPK*-ORF-R**	TCACATGTGCTGGTATTCAGGATTAAATGC
***NbWIPK*-ORF-F**	ATGGCTGATGCAAATATGGGTGGTGGT
***NbWIPK*-ORF-R**	TTAAGCATATTCAGGATTCAGCGACAAAGCTTCCTG
***NbSGT1*-ORF-F**	ATGGCGTCCGATCTGGAGACTA
***NbSGT1*-ORF-R**	CTAGATCTCCCATTTCTTCAGCTCCATGCC
***NbAOX1a*-ORF-F**	ATGACACGTGGAGCGACAAGGATGAC
***NbAOX1a*-ORF-R**	TTAGTGATACCCAATTGGTGCTGGAGAGTC
***NbAOX1b*-ORF-F**	ATGTGGGTTAGGCATTTTCCAGTG
***NbAOX1b*-ORF-R**	TTAGTGATACCCAATTGGTGCTGGAGAG

***NbMEK2*-ORF-F**	ATGCGACCTCTTCAACCACCCC
***NbMEK2*-ORF-R**	TCAAGAAGAAAAATGAGGCGGGGGTAAC
***NbNTF6*-ORF-F**	GTGAGACAGACAAAGAGAGCAGAA
***NbNTF6*-ORF-R**	CGATGAAACCAGCAATTTTGAGG
***NbRdRp1m*-ORF-F**	ATGGGAAAGACAATTCAGGTTTTTGGATTCCC
***NbRdRp1m*-ORF-R**	TCATCGTTCGAACGAGGTCAAACTAATGTCATCATTTGC
***NbSDE1*-ORF-F**	CATGGGATCAGAGGGCTCTGAAAAGGAA
***NbSDE1*-ORF-R**	TCATATATTGTCAACAAGATACCTTCCAAGGG
***NbERF3*-ORF-F**	GATGGCTGTCAAAAATAAGGTTAGTAATGGC
***NbERF3*-ORF-R**	TCAAAATTCCATAGGTGGAGCAAGGTTAAGATC
***NbPBS1*-ORF-F**	ATGGGTTGCTTCTCCTGTTTTGATTCAG
***NbPBS1*-ORF-R**	CTATCCATTAGTGCCATCAAAACTGCC
***NbICS*-ORF-F**	ATGGCTGTTGGTGCAAAGCACTGCACTC
***NbICS*-ORF-R**	TCAGAATGAGTGGTGTAGACAAACCAAAATGTGATGCTGC
***NbMEK1*-ORF-F**	ATGAAGACGACGAAGCCATTGAAGGAA
***NbMEK1*-ORF-R**	TATCTTGGAAAATTTACAGG
***NbRAR1*-ORF-F**	ATGGAGAGACTTCGTTGCCAGAGGATCGGT
***NbRAR1*-ORF-R**	TTAGGACGCTGGGCTGG
***NbWRKY13*-ORF-F**	ATGGATTTTGGGTTCAAGCAGAACCGACCGT
***NbWRKY13*-ORF-R**	TCATGGAACCTGTTCTTCTTTGAACCGTAGCAGTG
***NbWRKY12*-ORF-F**	ATGGCTGAGAATGAAGGATCATCATCATCATCGTCAAC
***NbWRKY12*-ORF-R**	TCATGTAATTTGTTCTTCTTTGAAACGTAGAAGGGC
***Nbcdpk2*-ORF-F**	ATGGGGAACACTTGTGTTGGGCCAAGTATTTCTAAAATTG
***Nbcdpk2*-ORF-R**	CTAAAGTCTTAGAGCCTCTCTAAATCCGGTGCTGAAACTG
***NbCOI1*-ORF-F**	ATGCCCATTCTCTTTCCTATTGCTTCTCGTCTGAC
***NbCOI1*-ORF-R**	CTATTCAGCGGGAAGGAAATTTCGGTCCAAAGG
***NbCTR1*-ORF-F**	ATGCTAAACTCAATGAACGTGGGCAAGGAC
***NbCTR1*-ORF-R**	TCAAATTACTCCATGGTTGTTGTAGTGTTGCCAACTCC
***NbWRKY3*-ORF-F**	ATGAAGAAGCGATTAGTTCATCAAAACCCACG

***NbWRKY3*-ORF-R**	TCAAGAAAACTCAATGGCCTCATCAAAGTG
***NbWRKY2*-ORF-F**	ATGAAAGAAGCATCTGATGTCTCTCTGTCTGATCAG
***NbWRKY2*-ORF-R**	TCATGGAACCTGTTCTTCTTTGAACCGTAGCAG
***NbWRKY1*-ORF-F**	ATGAGACAACCAGACATTCCAATGCTCACTGC
***NbWRKY1*-ORF-R**	TCAATTTGCACCAATATGAACAACCACGTC
***NbPR1a*-ORF-F**	ATGGGATTTGTTCTCTTTTCACAAATGC
***NbPR1a*-ORF-R**	TTAGTATGGACTTTGGCCTATATAATTACCTGGAGG
***NbEREBP1*-ORF-F**	ATGAATCAACCTATTTCTACAGAGTTGCCGCCG
***NbEREBP1*-ORF-R**	CCTAACTGACTAATAGTTGATGTCCACGTGTC
***NbERF5*-ORF-F**	ATGCGGAGAGGTAGAGCAGCGGC
***NbERF5*-ORF-R**	TCAAAGACAAAGCGCTGTGCAGTGAAGATCGT
***NbPAD4*-ORF-F**	ATGGTAGAAATAATGAACGCGAGCAAGTC
***NbPAD4*-ORF-R**	CTAAGGAACCATTTCAACCTCAAGAATTCCAGG
***NbTpoxC1*-ORF-F**	ATGGATCATTACTCCTCGATGACAC
***NbTpoxC1*-ORF-R**	CTAAATTATTGGTCTCGTTGTATATACGT
***NbNPK1*-ORF-F**	ATGCAGGATTTCATCGGCTCTGTTCGC
***NbNPK1*-ORF-R**	TTATCTTCCAGGAGATGCAAACCTTG
***NbNPR1*-ORF-F**	ATGGATAATAGTAGGACTGCGTTTTCTGATTCG
***NbNPR1*-ORF-R**	CTATTTCCTAAAAGGGAGCTTATTGGGCTTATCTACTC
***NbMYB1*-ORF-F**	ATGGGGAGAGCTCCTTGTTGTGAGAAAATGGGG
***NbMYB1*-ORF-R**	TCAAAATTCTGGTAATTCTGGCAAGTCATCAGTC
***NbCYBP*-ORF-F**	ATGGCAGCAAACGATTTAGCAATGG
***NbCYBP*-ORF-R**	TCAACTGAATCTCTTCAATGGATCAGCTG
***NbNUDC*-ORF-F**	TGACAAGAAGGGCCCTCGAGCT
***NbNUDC*-ORF-R**	TTAGTCCAACTTTAAGACGGTTC
***NbHSP20*-ORF-F**	ATGAGTAGTCGTCAACCAGAAGTTC
***NbHSP20*-ORF-R**	CTAATTTCCTCTAGCCTTTTCCAG
***NbNRG1*-pCambia1300-1 F**	TTGGAGAGAACACGGGGGACGAGCTCATGGCCGCAACTCTGT TGGGA

***NbNRG1*-pCambia1300-2 F**	CGATTTGTTGGATCCCGGGTACC TTAAGTAAAACTAAT
***NbNRG1*-pCambia1300-R**	CGATTTGTTGGATCCCGGGTACC TTAGATAAACAATGATTTGTG
***NbARF1*-pCambia1300-F**	TTGGAGAGAACACGGGGGACGAGCTCATGGGGCTAACATTC ACCAAG
***NbARF1*-pCambia1300-R**	CGATTTGTTGGATCCCGGGTACC TTAGGACTTGTTGGCAATGTT
***NbEDS1*-pCambia1300-F**	TTGGAGAGAACACGGGGGACGAGCTCATGGTGAGAATTGAA GAGGGG
***NbEDS1*-pCambia1300-R**	CGATTTGTTGGATCCCGGGTACC CTAAGAATTTACTTTCCCTGA
***NbHSP90*-pCambia1300-F**	TTGGAGAGAACACGGGGGACGAGCTCATGGCGGAGGCAGAG ACGTTT
***NbHSP90*-pCambia1300-R**	CGATTTGTTGGATCCCGGGTACC TTAGTCAACTTCCTCCATCTT
***NbSIPK*-pCambia1300-F**	TTGGAGAGAACACGGGGGACGAGCTCATGGATGGTTCTGGTC AGCA
***NbSIPK*-pCambia1300-R**	CGATTTGTTGGATCCCGGGTACC TCACATGTGCTGGTATTCAG
***NbWIPK*-pCambia1300-F**	TTGGAGAGAACACGGGGGACGAGCTCATGGCTGATGCAAAT ATGGG
***NbWIPK*-pCambia1300-R**	CGATTTGTTGGATCCCGGGTACC TTAAGCATATTCAGGATTCA
***NbSGT1*-pCambia1300-F**	TTGGAGAGAACACGGGGGACGAGCTCATGGCGTCCGATCTGG AGACTA
***NbSGT1*-pCambia1300-R**	CGATTTGTTGGATCCCGGGTACC CTAGATCTCCCATTTCTTCAG
***NbAOX1a*-pCambia1300-F**	TTGGAGAGAACACGGGGGACGAGCTCATGACACGTGGAGCG ACAAG

***NbAOX1a*-pCambia1300-R**	CGATTTGTTGGATCCCGGGTACCTTAGTGATACCCAATTGGTGCT
***NbAOX1b*-pCambia1300-F**	TTGGAGAGAACACGGGGGACGAGCTCATGTGGGTTAGGCATTTTCCA
***NbAOX1b*-pCambia1300-R**	CGATTTGTTGGATCCCGGGTACC TTAGTGATACCCAATTGGTGC
***NbMEK2*-pCambia1300-F**	TTGGAGAGAACACGGGGGACGAGCTCATGCGACCTCTTCAACCACC
***NbMEK2*-pCambia1300-R**	CGATTTGTTGGATCCCGGGTACCTCAAGAAGAAAAATGAGGCGG
***NbNTF6*-pCambia1300-F**	TTGGAGAGAACACGGGGGACGAGCTCATGGAAAACGAAACCAATGAA
***NbNTF6*-pCambia1300-R**	CGATTTGTTGGATCCCGGGTACC TCACTTCATTGTATTAGGATC
***NbRdRp1m*-pCambia1300-F**	TTGGAGAGAACACGGGGGACGAGCTCATGGGAAAGACAATTCAGGTT
***NbRdRp1m*-pCambia1300-R**	CGATTTGTTGGATCCCGGGTACCTCATCGTTCGAACGAGGTCAA
***NbSDE1*-pCambia1300-F**	TTGGAGAGAACACGGGGGACGAGCTCATGGGATCAGAGGGCTCTGAAAA
***NbSDE1*-pCambia1300-R**	CGATTTGTTGGATCCCGGGTACC TCATATATTGTCAACAAGA
***NbERF3*-pCambia1300-F**	TTGGAGAGAACACGGGGGACGAGCTCATGGCTGTCAAAAATAAGGTT
***NbERF3*-pCambia1300-R**	CGATTTGTTGGATCCCGGGTACCTCAAAACTCCGTAGGTGGAGC
***NbPBS1*-pCambia1300-F**	TTGGAGAGAACACGGGGGACGAGCTCATGGGTTGCTTCTCCTGTTTT
***NbPBS1*-pCambia1300-R**	CGATTTGTTGGATCCCGGGTACC CTATCCATTAGTGCCATCAAA

***NbICS*-pCambia1300-F**	TTGGAGAGAACACGGGGGACGAGCTCATGGCTGTTGGTGCAAAGCA
***NbICS*-pCambia1300-R**	CGATTTGTTGGATCCCGGGTACCTCAGAATGAGTGGTGTAGAC
***NbMEK1*-pCambia1300-F**	TTGGAGAGAACACGGGGGACGAGCTCATGAAGACGACGAAGCCATT
***NbMEK1*-pCambia1300-R**	CGATTTGTTGGATCCCGGGTACCTTATCTTGGAAAATTTACAGG
***NbRAR1*-pCambia1300-F**	TTGGAGAGAACACGGGGGACGAGCTCATGGAGAGACTTCGTTGCCAG
***NbRAR1*-pCambia1300-R**	CGATTTGTTGGATCCCGGGTACCTTAGGACGCTGGGCTGGCGTT
***NbWRKY13*-pCambia1300-F**	TTGGAGAGAACACGGGGGACGAGCTCATGGATTTTGGGTTCAAGCAG
***NbWRKY13*-pCambia1300-R**	CGATTTGTTGGATCCCGGGTACCTCATGGAACCTGTTCTTCTTT
***NbWRKY12*-pCambia1300-F**	TTGGAGAGAACACGGGGGACGAGCTCATGGCTGAGAATGAAGGATCA
***NbWRKY12*-pCambia1300-R**	CGATTTGTTGGATCCCGGGTACCTCATGTAATTTGTTCTTCTTTG
***Nbcdpk2*-pCambia1300-F**	TTGGAGAGAACACGGGGGACGAGCTCATGGGGAACACTTGTGTTGGG
***Nbcdpk2*-pCambia1300-R**	CGATTTGTTGGATCCCGGGTACCCTAAAGTCTTAGAGCCTCTCT
***NbCOI1*-pCambia1300-F**	TTGGAGAGAACACGGGGGACGAGCTCATGCCCATTCTCTTTCCTATT
***NbCOI1*-pCambia1300-R**	CGATTTGTTGGATCCCGGGTACCCTATTCAGCGGGAAGGAAAT
***NbCTR1*-pCambia1300-F**	TTGGAGAGAACACGGGGGACGAGCTCATGCTAAACTCAATG

	AACGTG
***NbCTR1*-pCambia1300-R**	CGATTTGTTGGATCCCGGGTACCTCAAATTACTCCATGGTTGTTG
***NbWRKY3*-pCambia1300-F**	TTGGAGAGAACACGGGGGACGAGCTCATGAAGAAGCGATTAGTTCAT
***NbWRKY3*-pCambia1300-R**	CGATTTGTTGGATCCCGGGTACCTCAAGAAAACTCAATGGCCTC
***NbWRKY2*-pCambia1300-F**	TTGGAGAGAACACGGGGGACGAGCTCATGAAAGAAGCATCTGATGTC
***NbWRKY2*-pCambia1300-R**	CGATTTGTTGGATCCCGGGTACCTCATGGAACCTGTTCTTCTTTG
***NbWRKY1*-pCambia1300-F**	TTGGAGAGAACACGGGGGACGAGCTCATGAGACAACCAGACATTCCA
***NbWRKY1*-pCambia1300-R**	CGATTTGTTGGATCCCGGGTACC TCAATTTGCACCAATATGAAC
***NbPR1a*-pCambia1300-F**	TTGGAGAGAACACGGGGGACGAGCTCATGGGATTTGTTCTCTTTTC
***NbPR1a*-pCambia1300-R**	CGATTTGTTGGATCCCGGGTACC TTAGTATGGACTTTGGCCTA
***NbEREBP1*-pCambia1300-F**	TTGGAGAGAACACGGGGGACGAGCTCATGAATCATTCTATTTATACA
***NbEREBP1*-pCambia1300-R**	CGATTTGTTGGATCCCGGGTACC TTAACTGACTATTAATTGATG
***NbERF5*-pCambia1300-F**	TTGGAGAGAACACGGGGGACGAGCTCATGCGGAGAGGTAGAGCAGG
***NbERF5*-pCambia1300-R**	CGATTTGTTGGATCCCGGGTACCTCAAAGACAAAGCGCTGTGCA
***NbPAD4*-pCambia1300-F**	TTGGAGAGAACACGGGGGACGAGCTCATGGTAGAAATAATGAACGCG

***NbPAD4*-pCambia1300-R**	CGATTTGTTGGATCCCGGGTACC CTAAGGAACCATTTCAACCTC
***NbTpoxC1*-pCambia1300-F**	TTGGAGAGAACACGGGGGACGAGCTCATGGCTTCTTTAAAGA TTAAT
***NbTpoxC1*-pCambia1300-R**	CGATTTGTTGGATCCCGGGTACC TTAATTGAGCCTACGGCAAT
***NbNPK1*-pCambia1300-F**	TTGGAGAGAACACGGGGGACGAGCTCATGCAGGATTTCATCG GCTCT
***NbNPK1*-pCambia1300-R**	CGATTTGTTGGATCCCGGGTACC TTATCTTCCAGGAGATGCAAA
***NbNPR1*-pCambia1300-F**	TTGGAGAGAACACGGGGGACGAGCTCATGGATAATAGTGGG ACTGCG
***NbNPR1*-pCambia1300-R**	CGATTTGTTGGATCCCGGGTACC CTATTTCCTAAAAGGGAGCTT
***NbMYB1*-pCambia1300-F**	TTGGAGAGAACACGGGGGACGAGCTCATGGGGAGAGCTCCT TGTTGT
***NbMYB1*-pCambia1300-R**	CGATTTGTTGGATCCCGGGTACC TCAAAATTCTGGTAATTCTGG
***NbCYBP*-pCambia1300-F**	TTGGAGAGAACACGGGGGACGAGCTCATGGCAGCAAACGAT TTAGCA
***NbCYBP*-pCambia1300-R**	CGATTTGTTGGATCCCGGGTACC TCAACTGAATCTCTTCAATGG
***NbNUDC*-pCambia1300-F**	TTGGAGAGAACACGGGGGACGAGCTCTGACAAGAAGGGCCC TCGAGCT
***NbNUDC*-pCambia1300-R**	CGATTTGTTGGATCCCGGGTACCTTAGTCCAACTTTAAGACGG TTC
***NbHSP20*-pCambia1300-F**	TTGGAGAGAACACGGGGGACGAGCTCATGAGTAGTCGTCAA CCAGAAGTTC
***NbHSP20*-pCambia1300-R**	CGATTTGTTGGATCCCGGGTACCCTAATTTCCTCTAGCCTTTT

	CCAG
***NbARF1*-RNAi-F**	TGTTGAGTACAAGAATATCAGC
***NbARF1*-RNAi-R**	ATGTACCAGTGACGCTGC
***NbCOI1*-RNAi-F**	CGTTGGGGAATCCGATGAAG
***NbCOI1*-RNAi-R**	TAGGCAAGTATATGGGCTGGATG
***NbPBS1*-RNAi-F**	ATCACTGGGCGTAAGGCTATTGACA
***NbPBS1*-RNAi-R**	ATTCCTGCATATTCTCCCACCTC
***NbSGT1*-RNAi-F**	CTAAAGATGCTCAACCAACTGTCAA
***NbSGT1*-RNAi-R**	AGGGTTCAGCTTTTGCAA
***NbARF1*-pQBV3-F**	CTTTGTACAAAAAAGCAGGCTCAGGGGATGATATCTGTTGAG TACAAGAATATCAGC
***NbARF1*-pQBV3-R**	TTGTACAAGAAAGCTGGGTGCAGGGCGATGATATCATGTACC AGTGACGCTGC
***NbCOI1*-pQBV3-F**	CTTTGTACAAAAAAGCAGGCTCAGGGGATGATATCCGTTGGG GAATCCGATGAAG
***NbCOI1*-pQBV3-R**	TTGTACAAGAAAGCTGGGTGCAGGGCGATGATATCTAGGCAA GTATATGGGCTGGATG
***NbPBS1*-pQBV3-F**	CTTTGTACAAAAAAGCAGGCTCAGGGGATGATATCATCACTG GGCGTAAGGCTATTGACA
***NbPBS1*-pQBV3-R**	TTGTACAAGAAAGCTGGGTGCAGGGCGATGATATCATTCCTG CATATTCTCCCACCTC
***NbSGT1*-pQBV3-F**	CTTTGTACAAAAAAGCAGGCTCAGGGGATGATATCCTAAAGA TGCTCAACCAACTGTCAA
***NbSGT1*-pQBV3-R**	TTGTACAAGAAAGCTGGGTGCAGGGCGATGATATCAGGGTTC AGCTTTTGCAA
***NbICS*-qPCR-F**	GTGAGTCATCATCACTCGT
***NbICS*-qPCR-R**	TTGGCACCTCAAGACGAATG
***NbMEK1*-qPCR-F**	GAACCACAAAGGAGAAGTTA
***NbMEK1*-qPCR-R**	GAAAATTGATCTGCTGGAG

***NbERF5*-qPCR-F**	CCTTATGCTCATCATCACCAC
***NbERF5*-qPCR-R**	ATCATTTTCCTTTTCACAGTCGC
***NbAOX1*-qPCR-F**	GAACCGGAGTTATGAGTGGC
***NbAOX1*-qPCR-R**	TCTCCCATGGCCTGAAGCAA
***NbTpoxC1*-qPCR-F**	GGCCTTATCTGGGGCTCA
***NbTpoxC1*-qPCR-R**	CTAGGATTGTTGCTGTAAGATGT
***NbSDE1*-qPCR-F**	ACTGGTTAGTAAAGATGAC
***NbSDE1*-qPCR-R**	CAATATCATCATCAGCAGTTC
